# Comprehensive clinical studies in 34 patients with molecularly defined UPD(14)pat and related conditions (Kagami–Ogata syndrome)

**DOI:** 10.1038/ejhg.2015.13

**Published:** 2015-02-18

**Authors:** Masayo Kagami, Kenji Kurosawa, Osamu Miyazaki, Fumitoshi Ishino, Kentaro Matsuoka, Tsutomu Ogata

**Affiliations:** 1 Department of Molecular Endocrinology, National Research Institute for Child Health and Development, Tokyo, Japan; 2 Division of Medical Genetics, Kanagawa Children's Medical Center, Yokohama, Japan; 3 Department of Radiology, National Center for Child Health and Development, Tokyo, Japan; 4 Department of Epigenetics, Medical Research Institute, Tokyo Medical and Dental University, Tokyo, Japan; 5 Department of Pathology, National Center for Child Health and Development, Tokyo, Japan; 6 Department of Pediatrics, Hamamatsu University School of Medicine, Hamamatsu, Japan

## Abstract

Paternal uniparental disomy 14 (UPD(14)pat) and epimutations and microdeletions affecting the maternally derived 14q32.2 imprinted region lead to a unique constellation of clinical features such as facial abnormalities, small bell-shaped thorax with a coat-hanger appearance of the ribs, abdominal wall defects, placentomegaly, and polyhydramnios. In this study, we performed comprehensive clinical studies in patients with UPD(14)pat (*n*=23), epimutations (*n*=5), and microdeletions (*n*=6), and revealed several notable findings. First, a unique facial appearance with full cheeks and a protruding philtrum and distinctive chest roentgenograms with increased coat-hanger angles to the ribs constituted the pathognomonic features from infancy through childhood. Second, birth size was well preserved, with a median birth length of ±0 SD (range, −1.7 to +3.0 SD) and a median birth weight of +2.3 SD (range, +0.1 to +8.8 SD). Third, developmental delay and/or intellectual disability was invariably present, with a median developmental/intellectual quotient of 55 (range, 29–70). Fourth, hepatoblastoma was identified in three infantile patients (8.8%), and histological examination in two patients showed a poorly differentiated embryonal hepatoblastoma with focal macrotrabecular lesions and well-differentiated hepatoblastoma, respectively. These findings suggest the necessity of an adequate support for developmental delay and periodical screening for hepatoblastoma in the affected patients, and some phenotypic overlap between UPD(14)pat and related conditions and Beckwith–Wiedemann syndrome. On the basis of our previous and present studies that have made a significant contribution to the clarification of underlying (epi)genetic factors and the definition of clinical findings, we propose the name ‘Kagami–Ogata syndrome' for UPD(14)pat and related conditions.

## Introduction

Human chromosome 14q32.2 carries a cluster of imprinted genes including paternally expressed genes (*PEGs*) such as *DLK1* and *RTL1,* and maternally expressed genes (*MEGs*) such as *MEG3* (alias, *GTL2*), *RTL1as* (*RTL1* antisense), *MEG8*, *snoRNAs*, and *microRNAs* (Supplementary Figure S1).^1, 2^ The parental origin-dependent expression patterns are regulated by the germline-derived primary *DLK1*-*MEG3* intergenic differentially methylated region (IG-DMR) and the postfertilization-derived secondary *MEG3*-DMR.^2, 3^ Both DMRs are hypermethylated after paternal transmission and hypomethylated after maternal transmission in the body; in the placenta, the IG-DMR alone remains as a DMR with the same methylation pattern in the body, while the *MEG3*-DMR does not represent a differentially methylated pattern.^2, 3^ Consistent with such methylation patterns, the hypomethylated IG-DMR and *MEG3*-DMR of maternal origin function as imprinting control centers in the placenta and the body, respectively, and the IG-DMR behaves hierarchically as an upstream regulator for the methylation pattern of the *MEG3*-DMR in the body, but not in the placenta.^3, 4^

Paternal uniparental disomy 14 (UPD(14)pat) (OMIM #608149) results in a unique constellation of clinical features such as facial abnormalities, small bell-shaped thorax with coat-hanger appearance of the ribs, abdominal wall defects, placentomegaly, and polyhydramnios.^2, 5^ These clinical features are also caused by epimutations (hypermethylations) and microdeletions affecting the maternally derived IG-DMR and/or *MEG3*-DMR (Supplementary Figure S1). Such UPD(14)pat and related conditions are rare, with reports of 33 patients with UPD(14)pat, five patients with epimutations, and nine patients with microdeletions (and four new UPD(14)pat patients reported here) (see Supplementary Table S1 for the reference list). For microdeletions, loss of the maternally inherited *MEG3*-DMR alone leads to a typical UPD(14)pat body phenotype and apparently normal placental phenotype,^3, 4^ whereas loss of the maternally derived IG-DMR alone or both DMRs results in a typical body and placental UPD(14)pat phenotype, consistent with the methylation patterns of the two DMRs.^2, 3^ Furthermore, correlations between clinical features and deleted segments have indicated the critical role of excessive *RTL1* (but not *DLK1*) expression in phenotypic development.^2, 6^ Such an excessive *RTL1* expression is primarily due to loss of functional *RTL1as*-encoded *microRNAs* that act as a *trans*-acting repressor for *RTL1* expression.^6^ Indeed, the *RTL1* expression level is ~5 times, rather than 2 times, increased in placentas with UPD(14)pat accompanied by two copies of functional *RTL1* and no functional *RTL1as*.^6^ This implies that the *RTL1* expression level is ~2.5 times increased in the absence of functional *RTL1as*-encoded *microRNAs*.

Here, we report comprehensive clinical findings in a series of patients with molecularly confirmed UPD(14)pat and related conditions, and suggest pathognomonic and/or characteristic features and their underlying factors. We also propose the name ‘Kagami–Ogata syndrome' for UPD(14)pat and related conditions.

## MATERIALS and methods

### Ethical approval

This study was approved by the Institute Review Board Committee at the National Center for Child Health and Development, and performed after obtaining written informed consent to publish the clinical and molecular information. We also obtained written informed consent with parental signature to publish facial photographs.

### Patients

This study consisted of 33 Japanese patients and one Irish patient (patient #32) with UPD(14)pat and related conditions (13 males and 21 females; 31 patients with normal karyotypes and two patients (#17 and #20) with Robertsonian translocations involving chromosome 14 (karyotyping not performed in patient #1); 30 previously described patients^2, 3, 7, 8, 9, 10^ and four new patients) in whom underlying (epi)genetic causes were clarified and detailed clinical findings were obtained (Supplementary Table S2).

The 34 patients were classified into three groups according to the underlying (epi)genetic causes that were determined by methylation analysis for the two DMRs, microsatellite analysis for a total of 24 loci widely dispersed on chromosome 14, fluorescence *in situ* hybridization for the two DMRs, and oligonucleotide array-based comparative genomic hybridization for the 14q32.2 imprinted region, as reported previously:^9^ (1) 23 patients with UPD(14)pat (UPD-group); (2) five patients with epimutations (Epi-group); and (3) six patients with microdeletions (Del-group) (Supplementary Figure S2).

Furthermore, the 23 patients of UPD-group were divided into three subtypes in terms of UPD generation mechanisms by microsatellite analysis, as reported previously:^9^ (1) 13 patients with monosomy rescue (MR) or postfertilization mitotic error (PE)-mediated UPD(14)pat indicated by full isodisomy (subtype 1) (UPD-S1); (2) a single patient with PE-mediated UPD(14)pat demonstrated by segmental isodisomy (subtype 2) (UPD-S2); and (3) nine patients with trisomy rescue (TR) or gamete complementation (GC)-mediated UPD(14)pat revealed by heterodisomy for at least one locus (subtype 3) (UPD-S3) (Supplementary Figure S2) (it is possible that some patients classified as UPD-S1 may have a cryptic heterodisomic region(s) and actually belong to UPD-S3). Similarly, the six patients of Del-group were divided into three subtypes in terms of the measured/predicted *RTL1* expression level in the body and placenta:^2, 3^ (1) three patients with ~5 times *RTL1* expression level in both the body and placenta (subtype 1) (Del-S1); (2) a single patient with about five times *RTL1* expression level in the body and normal (1 time) or ~2.5 times *RTL1* expression level in the placenta (subtype 2) (Del-S2); and (3) two patients with ~2.5 times *RTL1* expression level in both the body and placenta (subtype 3) (Del-S3) (Supplementary Figure S2). The measured/predicted expression patterns of the imprinted genes in each group/subtype are illustrated in Supplementary Figure S1.

### Clinical studies

We used a comprehensive questionnaire to collect detailed clinical data of all patients from attending physicians. To evaluate chest roentgenographic findings, we obtained the coat-hanger angle (CHA) to the ribs and the ratio of the mid to widest thorax diameter (M/W ratio), as reported previously.^11^ We also asked the physicians to report any clinical findings not covered by the questionnaire.

### Statistical analysis

Statistical significance of the median among three groups and between two groups/subtypes was examined by the Kruskal–Wallis test and the Mann–Whitney's *U*-test, respectively, and that of the frequency among three groups and between two groups was analyzed by the Fisher's exact probability test, using the R environment (http://cran.r-project.org/bin/windows/base/old/2.15.1/). *P*<0.05 was considered significant. Kaplan–Meier survival curves were constructed using the R environment.

## Results

Clinical findings of each group/subtype are summarized in Table 1, and those of each patient are shown in Supplementary Table S2. Phenotypic findings were comparable among UPD-S1, UPD-S2, and UPD-S3, and somewhat different among Del-S1, Del-S2, and Del-S3, as predicted from the expression patterns of the imprinted genes (Supplementary Figure S1). Thus, we showed the data of UPD-group (the sum of UPD-S1, UPD-S2, and UPD-S3) and those of each subtype of Del-group (Del-S1, Del-S2, and Del-S3) in Table 1, and described the data of UPD-S1, UPD-S2, and UPD-S3 in Supplementary Table S3.

We registered the clinical information of each patient in the Leiden Open Variation Database (LOVD) (http://www.lovd.nl/3.0/home; http://databases.lovd.nl/shared/individuals), and the details of each microdeletion in the ClinVar Database (http://www.ncbi.nlm.nih.gov/clinvar/). The LOVD Individual IDs and the ClinVar SCV accession numbers are shown in Supplementary Table S2.

### Pregnancy and delivery

Polyhydramnios was observed from ~25 weeks of gestation during the pregnancies of all patients, except for patient #32 of Del-S2 who had deletion of the *MEG3*-DMR and three of the seven *MEG3* exons, and usually required repeated amnioreduction, especially after 30 weeks of gestation. Placentomegaly was usually identified in patients affected with polyhydramnios, but not found in three patients of UPD-group. Thoracic and abdominal abnormalities were found by ultrasound studies in ~40% of patients from ~25 weeks of gestation, and UPD(14)pat was suspected in patients #7 and #21, due to delineation of the bell-shaped thorax with coat-hanger appearance of the ribs. Premature delivery was frequently observed, especially in Del-group. Because of fetal distress and polyhydramnios, ⩾two-thirds of the patients in each group were delivered by Cesarean section. Medically assisted reproduction was reported only in one (patient #8) of 21 patients for whom clinical records on conception were available.

### Growth pattern

Prenatal growth was characterized by grossly normal birth length and obviously excessive birth weight. Indeed, birth length ranged from 30.6 to 51.0 cm (−1.7 to +3.0 SD for the gestational age- and sex-matched Japanese reference data) with a median of 44.7 cm (±0 SD), and birth weight ranged from 1.24 to 3.77 kg (+0.1 to +8.8 SD) with a median of 2.79 kg (+2.3 SD). Although birth weight was disproportionately greater than birth length, there was no generalized edema as a possible cause of overweight.

In contrast, postnatal growth was rather compromised, and growth failure (present length/height and/or weight <−2 SD) was observed in about one-third of patients of each group. Postnatal weight was better preserved than postnatal length/height.

### Craniofaciocervical features

**Figure 1:**
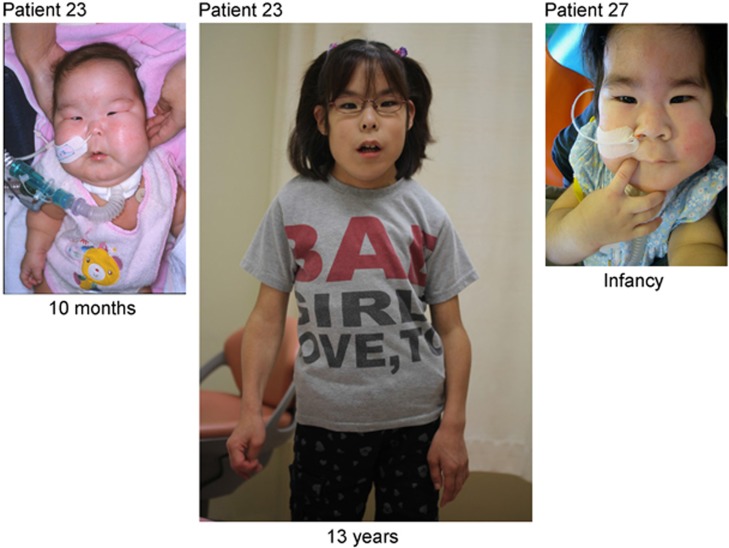
Photographs of patient #23 with UPD(14)pat and patient #27 with epimutation.

All patients exhibited strikingly similar craniofaciocervical features (Figure 1). Indeed, >90% of patients had depressed nasal bridge, full cheeks, protruding philtrum, micrognathia, and short webbed neck. In particular, the facial features with full cheeks and protruding philtrum appeared to be specific to UPD(14)pat and related conditions, and were recognizable from infancy through childhood.

### Thoracic abnormality

**Figure 2:**
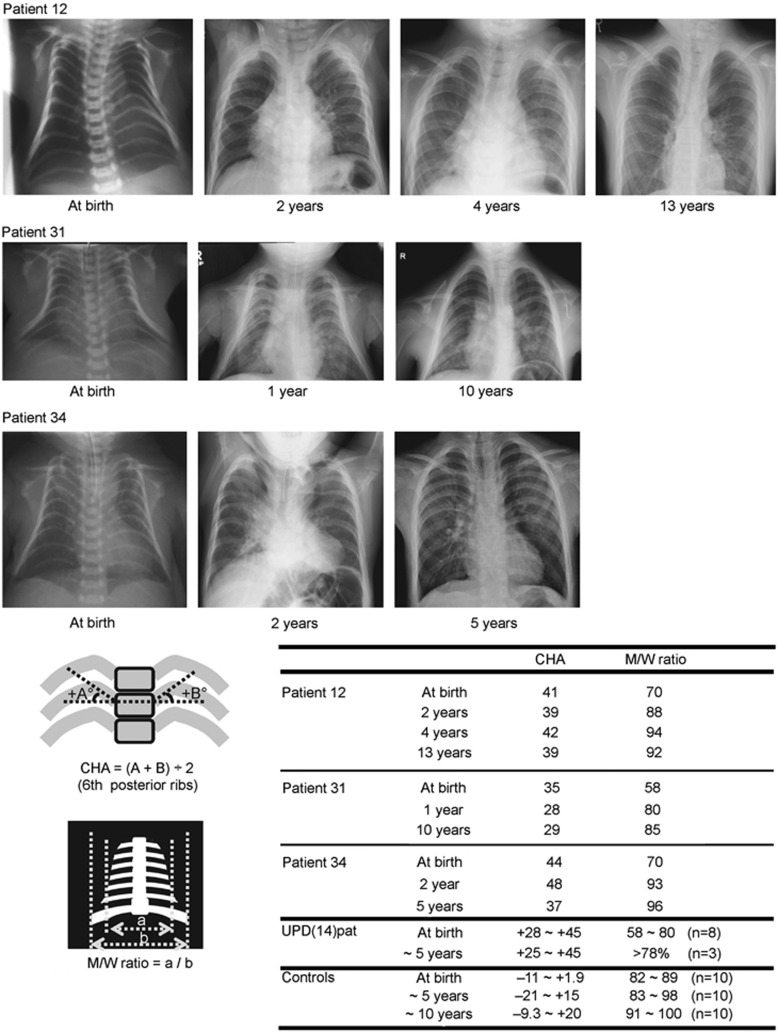
Chest roentgenograms of patient #12 of UPD-group, patient #31 of Del-S1, and patient #34 of Del-S3. *RTL1* expression level is predicted to be ~5 times higher in patients #12 and #31, and ~2.5 times higher in patient #34. The CHA to the ribs remains above the normal range throughout the study period, whereas the M/W ratio (the ratio of the mid to widest thorax diameter) normalizes with age.

The 34 patients invariably showed small bell-shaped small thorax with coat-hanger appearance of the ribs in infancy (Figure 2). Long-term (⩾10 years) follow-up in patient #12 of UPD-group and patient #31 of Del-S1 who had ~5 times of *RTL1* expression, and in patient #34 of Del-S3 who had ~2.5 times of *RTL1* expression, showed that the CHAs remained above the normal range of age-matched control children, while the M/W ratios, though they were below the normal range in infancy, became within the normal range after infancy (Figure 2). Laryngomalacia was also often detected in each group.

Mechanical ventilation was performed in all patients except for patients #14 and #20 of UPD-group, and tracheostomy was also carried out in about one-third of patients. Mechanical ventilation could be discontinued during infancy in 22 patients (Supplementary Figure S3). Ventilation duration was variable with a median period of 1 month among the 22 patients, and was apparently unrelated to the underlying genetic cause or gestational age.

### Abdominal wall defects

Omphalocele was identified in about one-third of patients, and diastasis recti was found in the remaining patients.

### Developmental status

**Figure 3:**
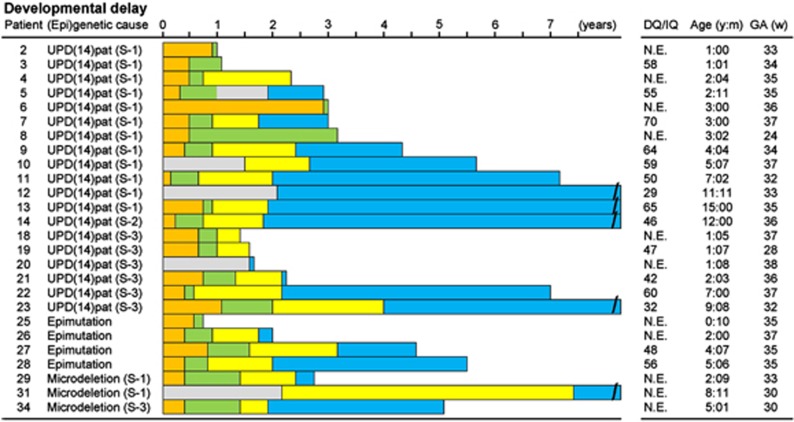
Developmental status. The orange, green, yellow, and blue bars represent the period before head control, the period after head control and before sitting without support, the period after sitting without support and before walking without support, and the period after walking without support, respectively. The gray bars denote the period with no information. DQ, developmental quotient; IQ, intellectual quotient; N.E., not examined; Age, age at the last examination or at death; and GA, gestational age.

Developmental delay (DD) and/or intellectual disability (ID) was invariably present in 26 patients examined (age, 10 months to 15 years), with the median developmental/intellectual quotient (DQ/IQ) of 55 (range, 29–70) (Figure 3). Gross motor development was also almost invariably delayed, with grossly similar patterns among different groups. In patients who passed gross motor developmental milestones, head control was achieved at ~7 months, sitting without support at ~12 months, and walking without support at ~2.1 years of age.

### Other features

Several prevalent features were also identified. In particular, except for patient #22, feeding difficulty with poor sucking and swallowing was exhibited by all patients who were affected with polyhydramnios, and gastric tube feeding was performed in all patients who survived more than 1 week (Supplementary Figure S4). Tube-feeding duration was variable with a median period of ~7.5 months in 16 patients for whom tube feeding was discontinued, and tended to be longer in Del-group. In addition, there were several features manifested by single patients (Supplementary Table S2).

**Figure 4:**
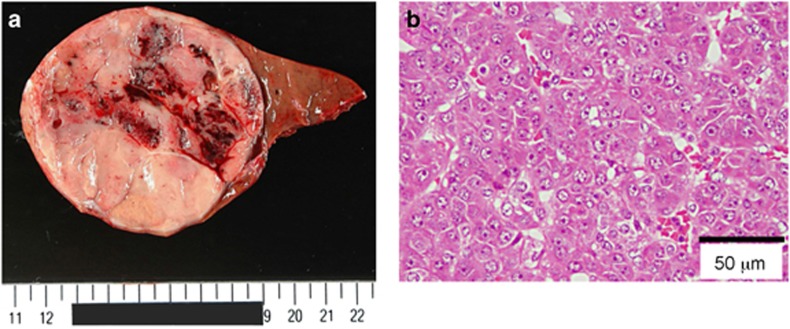
Hepatoblastoma in patient #8 of UPD-group. (**a**) Macroscopic appearance of the hepatoblastoma with a diameter of ~8 cm. (**b**) Microscopic appearance of the hepatoblastoma exhibiting a trabecular pattern. The hepatoblastoma cells are associated with eosinophilic cytoplasm and large nuclei, and resemble fetal hepatocytes.

Notably, hepatoblastoma was identified at 46 days of age in patient #17, at 218 days in patient #18, and at 13 months of age in patient #8 of UPD-group (Figure 4). It was surgically removed in patients #8 and #18, although chemotherapy was not performed because of poor body condition. In patient #17, neither an operation nor chemotherapy could be carried out because of the patient's severely poor body condition. Histological examination of the removed tumors revealed a poorly differentiated embryonal hepatoblastoma with focal macrotrabecular lesions in patient #8 (Figure 4) and a well-differentiated hepatoblastoma in patient #18.^10^

### Mortality

**Figure 5:**
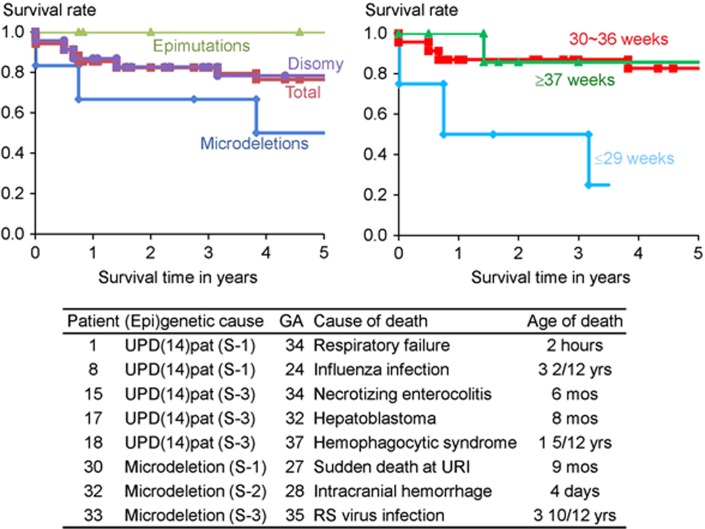
Kaplan–Meier survival curves according to the (epi)genetic cause and the gestational age (week), and summary of the causes of death. GA, gestational age; URI, upper respiratory infection; and RS, respiratory syncytial. Patients #8, #17, and #18 had hepatoblastoma.

Eight patients were deceased before 4 years of age. The survival rate was 78% in UPD-group, 100% in Epi-group, and 50% in Del-group; it was 25% in patients born ⩽29 weeks of gestation, 83% in those born 30–36 weeks of gestation, and 86% in those born ⩾37 weeks of gestation (Figure 5). The cause of death was variable; however, respiratory problems were a major factor, because patient #1 died of neonatal respiratory distress syndrome, and patients #8, #30 and #33 died during a respiratory infection. Of the three patients with hepatoblastoma, patient #17 died of hepatoblastoma, whereas patient #8 died during influenza infection and patient #18 died of hemophagocytic syndrome.

### Comparison among/between different groups/subtypes

Clinical findings were grossly similar among/between different groups/subtypes with different expression dosages of *RTL1* and *DLK1*. However, significant differences were found for short gestational age and long duration of tube feeding in Del-group (among three groups and against Epi-group and UPD-group) and infrequent hairy forehead in Epi-group (among three groups and against UPD-group) (actual *P*-values are available on request).

## Discussion

We examined detailed clinical findings in patients with UPD(14)pat and related conditions. The results indicate that the facial features with full cheeks and protruding philtrum and the thoracic roentgenographic findings with increased CHAs to the ribs represent the pathognomonic features of UPD(14)pat and related conditions from infancy through the childhood. In addition, the decreased M/W ratios also denote the diagnostic hallmark in infancy, but not after infancy. Although other features such as polyhydramnios, placentomegaly, and abdominal wall defects are characteristic of UPD(14)pat and related conditions, they would be regarded as rather nonspecific features that are also observed in other conditions such as Beckwith–Wiedemann syndrome (BWS) (Supplementary Table S4).^12, 13^

Such body and placental features were similarly exhibited by patients of each group/subtype, including those of Del-S1, Del-S2, and Del-S3 with different expression dosage of *DLK1* (1 × or 2 × ) and *RTL1* (~2.5 × or ~5 × ), except for patient #32 of Del-S2 who showed typical body features but apparently lacked placental features. Indeed, the difference in the *DLK1* expression dosage had no discernible clinical effects, although mouse *Dlk1* is expressed in several fetal tissues, including the ribs.^14, 15^ Similarly, in contrast to our previous report which suggested a possible dosage effect of *RTL1* expression level on the phenotypic severity,^2^ the difference in the *RTL1* expression dosage turned out to have no recognizable clinical effects after analyzing long-term clinical courses in the affected patients. This suggests that ~2.5 × *RTL1* expression is the primary factor for the phenotypic development in the body and placenta. Consistent with the critical role of excessive *RTL1* expression in the phenotypic development, mouse *Rtl1* is clearly expressed in the fetal ribs and skeletal muscles (Supplementary Figure S5) as well as in the placenta,^16, 17^ and human *RTL1* mRNA and RTL1 protein are strongly expressed in placentas with UPD(14)pat.^6^ Thus, lack of placental abnormalities in patient #32 can be explained by assuming a positive *RTL1as* expression and resultant normal (1 × ) *RTL1* expression in the placenta (Supplementary Figure S1). In addition, since mouse *Gtl2* (*Meg3*) is expressed in multiple fetal tissues including the primordial cartilage,^14^ this may argue for the positive role of absent *MEGs* expression in phenotypic development.

The present study revealed several notable findings. First, polyhydramnios was identified during the pregnancies of nearly all patients, except for patient #32 of Del-S2. Amniotic fluid originates primarily from fetal urine and is absorpted primarily by fetal swallowing into the digestive system.^18, 19^ Since fetal hydration and the resultant urine flow mainly depend on the water flow from maternal circulation across the placenta,^19^ placentomegaly would have facilitated the production of amniotic fluid. Furthermore, since feeding difficulty with impaired swallowing was observed in most patients, defective swallowing would have compromised absorption of amniotic fluid. Thus, both body and placental factors are assumed for the development of polyhydramnios. This would explain why polyhydramnios was observed in patients #1, #6, and #8 who were free from placentomegaly, and in patient #22 who showed no feeding difficulty, although the presence of feeding difficulty was unknown for patient #1 as was placentomegaly for patient #22. In addition, since amniotic fluid begins to increase from 8–11 weeks of gestation and reaches its maximum volume around 32 weeks of gestation,^18, 19^ this would explain why amnioreduction was usually required from 30 weeks of gestation.

Second, birth size was relatively well preserved, whereas postnatal growth was rather compromised. The well preserved prenatal growth in apparently compromised intrauterine environments would be consistent with the conflict theory that overexpression of *PEGs* promotes fetal and placental growth.^20^ Notably, birth weight was disproportionately greater than birth length in the apparent absence of generalized edema. In this regard, mouse *Dlk1*, *Rtl1*, and *Gtl2* (*Meg3*) on the distal part of chromosome 12 are expressed in skeletal muscles (Supplementary Figure S5),^14, 17^ and paternal disomy for chromosome 12 causes muscular hypertrophy.^21^ Thus, patients with UPD(14)pat and related conditions may have muscular hypertrophy especially in the fetal life. The compromised postnatal growth would primarily be because of poor nutrition caused by feeding difficulties, whereas relative overweight suggestive of possible muscular hypertrophy remains to be recognized.

Third, DD/ID was invariably present in all 26 patients examined for their developmental/intellectual status, with the median DQ/IQ of 55. In this regard, mouse *Dlk1*, *Rtl1*, and *Gtl2* (*Meg3*) are expressed in the brain during embryogenesis (Supplementary Figure S5),^22^ and *Dlk1* is involved in the differentiation of midbrain dopaminergic neurons.^22^ Thus, DD/ID would primarily be ascribed to the altered expression dosage of *PEGs*/*MEGs* in the brain.

Fourth, hepatoblastoma was identified in three patients of UPD-group during infancy. In this context, it has been reported that (1) mouse *Dlk1*, *Rtl1*, and *Meg3* (*Gtl2*) are expressed in the fetal liver, but not in the adult liver; ^14, 17, 23, 24^ (2) overexpression of *Rtl1* in the adult mouse liver has induced hepatic tumors with high penetrance;^24^ (3) *Meg3* functions as a tumor suppressor gene in mice;^25^ (4) human *DLK1* is expressed in the hepatocytes of 5–6 weeks old embryos;^26^ and (5) human DLK1 protein is upregulated in hepatoblastoma.^27^ These findings imply the relevance of excessive *RTL1* expression and loss of *MEG3* expression to the occurrence of hepatoblastoma in UPD(14)pat and related conditions, while it remains to be determined whether the DLK1 upregulation is the cause or the result of hepatoblastoma development. Thus, periodical screening for hepatoblastoma, such as serum *α*-fetoprotein measurement and abdominal ultrasonography, is recommended. In this context, it remains to be studied whether other embryonal tumors may also be prone to occur in UPD(14)pat and related conditions.

Fifth, mortality was high in Del-group and null in Epi-group. The high mortality in Del-group would primarily be ascribed to the high prevalence of premature delivery, although it is unknown whether the high prevalence of premature delivery is an incidental finding or characteristic of Del-group. The null mortality in Epi-group may be due to possible mosaicism with cells accompanied by a normal expression pattern because of escape from epimutation, as reported previously.^28, 29^ It is unknown, however, whether possible presence of trisomic cells in TR-mediated UPD(14)pat and that of normal cells in PE-mediated UPD(14) may have exerted clinical effects. Notably, since death was observed only in patients <4 years of age, the vital prognosis is expected to be good from childhood. In addition, since three patients died during respiratory infections, careful management is recommended during such infections.

Furthermore, the present study also provides several useful clinical implications: (1) two patients had Robertsonian translocations as a risk factor for the development of UPD.^30^ Thus, karyotyping is suggested for patients with an UPD(14)pat-like phenotype; (2) prenatal detection of polyhydramnios and thoracic and abdominal features is possible from ~25 weeks of gestation; (3) mechanical ventilation and gastric tube feeding are usually required, with variable durations; (4) there was no patient in UPD-group who exhibited clinical features that are attributable to the unmasking of a recessive mutation(s) of paternal origin; (5) since UPD(14)pat and related conditions share several clinical features including embryonal tumors with BWS (Supplementary Table S4), UPD(14)pat and related conditions may be worth considering in atypical or underlying factor-unknown BWS; and (6) since clinical findings are comparable between patients examined in this study and 17 similarly affected non-Japanese patients (Supplementary Table S5), our data will be applicable to non-Japanese patients as well.

A critical matter for UPD(14)pat and related conditions is the lack of a syndrome name. Although the term ‘UPD(14)pat syndrome' has been utilized previously,^4^ the term is confusing because ‘UPD(14)pat syndrome' can be caused by (epi)genetic mechanisms other than UPD(14)pat. In this regard, the name ‘Temple syndrome' has been proposed for UPD(14)mat and related conditions or ‘UPD(14)mat syndrome',^31, 32^ a mirror image of UPD(14)pat and related conditions. On the basis of our previous and present studies that have made a significant contribution to the clarification of underlying (epi)genetic factors and the definition of clinical findings, we would propose the name ‘Kagami–Ogata syndrome', or ‘Wang–Kagami–Ogata syndrome' with the name of Wang who first described UPD(14)pat,^33^ for UPD(14)pat and related conditions.

In summary, although the number of patients still remains small, especially in each subtype of Del-group, the present study reveals pathognomic and characteristic clinical findings in UPD(14)pat and related conditions. Furthermore, this study shows the invariable occurrence of DD/ID and the occasional (8.8%) development of hepatoblastoma, thereby showing the necessity of adequate support for DD/ID and screening of hepatoblastoma in affected patients. Finally, we propose the name ‘Kagami–Ogata syndrome' for UPD(14)pat and related conditions.

## Figures and Tables

**Table 1:** Clinical manifestations in 33 Japanese and one Irish patients with UPD(14)pat and related conditions (Kagami–Ogata syndrome)

	*UPD(14)pat*	*Epimutations*	*Microdeletions*				*Total*
			*Subtype 1*	*Subtype 2*	*Subtype 3*	*Subtotal*	
	*Pts 1–23 (*n=*23)*	*Pts 24–28 (*n=*5)*	*Pts 29–31 (*n=*3)*	*Pt 32 (*n=*1)*	*Pts 33–34 (*n=*2)*	*Pts 29–34 (*n=*6)*	*Pts 1–34 (*n=*34)*
Age at the last examination or death (y)	2.9 (0.0–15.0)	2.0 (0.8–5.5)	2.8 (0.8–8.9)	(4 days)	4.5 (3.8–5.1)	3.3 (0.0–8.9)	2.8 (0.0–15.0)
Sex (male:female)	9:14	3:2	1:2	0:1	0:2	1:5	13:21
							
*Molecular findings*^a^							
IG-DMR of maternal origin	Absent	Methylated	Deleted	Unmethylated	Deleted		
*MEG3*-DMR of maternal origin	Absent	Methylated^b^	Deleted/methylated^b^	Deleted	Deleted		
*DLK1* expression level	2 ×	2 ×	1 or 2 ×	2 × (1 × )^c^	1 or 2 ×		
*RTL1* expression level	~5 ×	~5 ×	~5 ×	~5 × (1 × or ~2.5 × )^c^	~2.5 ×		
*MEGs* expression level	0 ×	0 ×	0 ×	0 × (1 × or 0 × )^c^	0 ×		
							
*Pregnancy and delivery*							
Polyhydramnios	23/23	5/5	3/3	0/1	2/2	5/6	33/34
Gestational age at Dx (w)	25 (14–30)	27.5 (22–30)	Unknown	—	21	21	25.5 (14–30)
Amnioreduction	18/20	4/5	2/3	0/1	1/2	3/6	25/31
Amnioreduction (>30 w)	18/18	4/4	2/2	—	1/1	3/3	25/25^d^
Placentomegaly^e^	14/17	4/4	3/3	0/1	2/2	5/6	23/27
Prenatal Dx of thoracic abnormality	8/20^f^	2/3	0/1	—	0/1	0/2	10/25
Gestational age at Dx (w)	26 (22–33)	27.5 (25–30)	—	—	—	—	26 (22–33)
Prenatal Dx of abdominal abnormality	6/18	3/3	1/1	—	0/1	1/2	10/23
Gestational age at Dx (w)	26 (22–28)	25	Unknown	Unknown	Unknown	Unknown	25.5 (22–28)
Gestational age (w)	34.5 (24–38)	35 (30–37)	30 (27–33)	28	32.5 (30–35)	30 (27–35)	34 (24–38)
Premature delivery (<37 w)	17/23	4/5	3/3	1/1	2/2	6/6	27/34
Delivery (Cesarean:Vaginal)	15:8	4:1	2:1	0:1	2:0	4:2	23:11
Medically assisted reproduction	1/18	0/1	0/1	Unknown	0/1	0/2	1/21
							
*Growth pattern*							
Prenatal growth failure^g^	0/23	0/5	0/3	0/1	0/2	0/6	0/34
Prenatal overgrowth^h^	13/23	3/5	3/3	0/1	1/2	4/6	20/34
Birth length (patient number)	21	5	1	1	2	4	30
SD score, median (range)	+0.3 (−1.7 to +3.0)	−0.5 (−0.9 to +1.4)	0.0	−1.1	+0.7 (−0.1 to +1.5)	−0.1 (−1.1 to +1.5)	±0 (−1.7 to +3.0)
Actual length (cm), median (range)	45.0 (30.6 to 51.0)	43.5 (41.0 to 50.0)	43.0	34.0	43.5 (42.0 to 45.0)	42.5 (34.0 to 45.0)	44.7 (30.6 to 51.0)
Birth weight (patient number)	23	5	3	1	2	6	34
SD score, median (range)	+2.2 (+0.1 +8.8)	+2.2 (+0.5 to +3.7)	+2.8 (+2.4 to +3.7)	+1.5	+1.7 (+0.9 to +2.5)	+2.5 (+0.9 to +3.7)	+2.3 (+0.1 to +8.8)
Actual weight (cm), median (range)	2.79 (1.24 to 3.77)	2.9 (1.61 to 3.28)	2.04 (1.30 to 2.84)	1.32	2.24 (1.55 to 2.94)	1.79 (1.30 to 2.94)	2.79 (1.24 to 3.77)
Postnatal growth failure^i^	7/20	2/5	2/3	—	0/2	2/5	11/30
Postnatal overgrowth^j^	1/20	1/5	0/3	—	0/2	0/5	2/30
Present stature (patient number)	20	5	3	—	1	4	29
SD score, median (range)	−1.6 (−8.7 to +1.1)	−1.8 (−7.1 to +0.9)	−2.2 (−3.3 to −1.3)	—	−1.6	−1.9 (−3.3 to −1.3)	−1.6 (−8.7 to +1.1)
Present weight (patient number)	20	5	3	—	2	5	30
SD score, median (range)	−1.0 (−6.0 to +2.4)	−0.6 (−5.5 to +4.0)	−1.3 (−2.2 to ±0)	—	−1.1 (−1.3 to −0.9)	−1.3 (−2.2 to ±0)	−1.0 (−6.0 to +4.0)
							
*Craniofaciocervical features*							
Frontal bossing	17/22	4/5	1/3	1/1	2/2	4/6	25/33
Hairy forehead	18/22	1/5	3/3	1/1	0/2	4/6	23/33
Blepharophimosis	18/22	3/5	2/3	0/1	1/2	3/6	24/33
Small ears	8/21	2/5	1/3	1/1	0/2	2/6	12/32
Depressed nasal bridge	23/23	5/5	3/3	0/1	1/2	4/6	32/34
Anteverted nares	19/22	4/5	3/3	0/1	2/2	5/6	28/33
Full cheek	20/21	4/4	2/2	0/1	1/1	3/4	27/29
Protruding philtrum	23/23	5/5	3/3	0/1	2/2	5/6	33/34
Puckered lips	11/21	3/5	3/3	0/1	0/2	3/6	17/32
Micrognathia	20/21	5/5	3/3	1/1	1/2	5/6	30/32
Short webbed neck	22/22	5/5	3/3	1/1	2/2	6/6	33/33
							
*Thoracic abnormality*							
Small bell-shaped thorax in infancy^k^	23/23	5/5	3/3	1/1	2/2	6/6	34/34
Coat-hanger appearance in infancy^l^	23/23	5/5	3/3	1/1	2/2	6/6	34/34
Laryngomalacia	8/20	2/5	2/3	—	0/1	2/4	12/29
Tracheostomy	7/21	1/4	0/2	—	2/2	2/4	10/29
Mechanical ventilation	21/23	5/5	3/3	1/1	2/2	6/6	32/34
Duration of ventilation (m)^m^	1.2 (0.1–17)	0.7 (0.1–0.9)	5 (0.23–10)	—	1.5 (1–2)	2 (0.2–10)	1.0 (0.1–17)
							
*Abdominal wall defects*							
Omphalocele	7/23	2/5	1/3	1/1	0/2	2/6	11/34
Diastasis recti	16/23	3/5	2/3	0/1	2/2	4/6	23/34
*Developmental delay*							
Developmental delay	21/21	5/5	3/3	—	2/2	5/5	31/31
Developmental/intellectual quotient	55 (29–70)	52 (48–56)	Unknown	Unknown	Unknown	—	55 (29–70)
Delayed head control (>4 m)^n^	14/16	4/4	1/1	—	1/1	2/2	20/22
Age at head control (m)^o^	7 (3–36)	7 (6–11)	6	—	6	6 (6)	7 (3–36)
Delayed sitting without support (>7 m)^n^	16/16	4/4	2/2	—	1/1	3/3	23/23
Age at sitting without support (m)^o^	12 (8–25)	11.5 (10–20)	22.5 (18–27)	—	18	18 (18–27)	12 (8–27)
Delayed walking without support (>14 m)^n^	17/17	3/3	2/2	—	2/2	4/4	24/24
Age at walking without support (m)^o^	25.5 (20–49)	25 (22–39)	60 (30–90)	—	24	30 (24–90)	25.5 (20–90)
							
*Other features*							
Feeding difficulty	20/21	5/5	3/3	—	2/2	5/5	30/31
Duration of tube feeding (m)^p^	6 (0.1–72)	8.5 (0.5–17)	59.5 (30–89)	—	51	51 (30–89)	7.5 (0.1–89)
Joint contractures	14/22	3/5	3/3	0/1	0/2	3/6	20/33
Constipation	12/20	3/4	1/2	—	0/2	1/4	16/28
Kyphoscoliosis	9/21	3/5	1/2	0/1	0/1	1/4	13/30
Coxa valga	6/21	1/5	3/3	0/1	1/2	4/6	11/32
Cardiac disease	5/22	1/5	0/3	1/1	1/2	2/6	8/33
Inguinal hernia	5/22	1/5	2/3	0/1	0/2	2/6	8/33
Seizure	1/21	0/5	0/3	0/1	0/2	0/6	1/32
Hepatoblastoma	3/23	0/5	0/3	0/1	0/2	0/6	3/34
							
*Mortality within the first 5 years*							
Alive:deceased	18:5	5:0	2:1	0:1	1:1	3:3	26:8
*Parents*							
Paternal age at childbirth (y)	35 (24–47)	30 (26–36)	37 (34–39)	25	31.5 (27–36)	35 (25–39)	34 (24–47)
Maternal age at childbirth (y)	31 (25–43)	28 (25–35)	31 (27–36)	25	30.5 (28–33)	29.5 (25–36)	31 (25–43)
Advanced childbearing age (⩾35 y)	8/23	1/5	1/3	0/1	0/2	1/6	8/34

Abbreviations: CHA, coat-hanger angle; Dx, diagnosis; m, month; M/W, mid to widest thorax diameter; UPD(14)pat, paternal uniparental disomy 14; w, week; y, year.

Patient #32 is Irish, and the remaining patients are Japanese; the Irish patient has also been examined by Beygo *et al.*^4^

Age data are expressed by median and range.

The denominators indicate the number of patients examined for the presence or absence of each feature, and the numerators represent the number of patient assessed to be positive for that feature; thus, differences between the denominators and numeratorsdenote the number of patients evaluated to be negative for the feature.

a For details, see Supplementary Figures S1 and S2.

b The *MEG3*-DMR is predicted to be grossly hypomethylated in the placenta.

c Expression patterns of the imprinted genes are predicted to be different between the body and the placenta in this patient, while they are predicted to be identical between the body and the placenta in other patients (See Supplementary Figure S1).

d Amnioreduction was performed about two times in 23 of the 25 pregnancies.

e Placental weight >120% of the gestational age-matched mean placental weight.^34^

f The diagnosis of UPD(14)pat has been suspected in two patients (patients #7 and #21).

g Birth length and/or birth weight <−2 SD of the gestational age- and sex-matched Japanese reference data (http://jspe.umin.jp/medical/keisan.html).

h Birth length and/or birth weight >+2 SD of the gestational age- and sex-matched Japanese reference data (http://jspe.umin.jp/medical/keisan.html).

i Present length/height and/or present weight <−2 SD of the age- and sex-matched Japanese reference data (http://jspe.umin.jp/medical/taikaku.html).

j Present length/height and/or present weight >+2 SD of the age- and sex-matched Japanese reference data (http://jspe.umin.jp/medical/taikaku.html).

k The M/W ratio below nromal range (see Figure 2).

l The CHA above the normal range (see Figure 2).

m The duration in patients in whom mechanical ventilation could be discontinued.

n The age when 90% of infants pass each gross motor developmental milestone (based on Revised Japanese Version of Denver Developmental Screening Test) (http://www.dinf.ne.jp/doc/japanese/prdl/jsrd/norma/n175/img/n175_078i01.gif).

o The median (range) of ages in patients who passed each gross motor developmental milestone; patients who have not passed each milestone are not included.

p The duration in patients in whom tube feeding could be discontinued.

## References

[bib1] 1da Rocha ST, Edwards CA, Ito M, Ogata T, Ferguson-Smith AC: Genomic imprinting at the mammalian Dlk1-Dio3 domain. Trends Genet 2008; 24: 306–316. 18471925 10.1016/j.tig.2008.03.011

[bib2] 2Kagami M, Sekita Y, Nishimura G et al: Deletions and epimutations affecting the human 14q32.2 imprinted region in individuals with paternal and maternal upd(14)-like phenotypes. Nat Genet 2008; 40: 237–242. 18176563 10.1038/ng.2007.56

[bib3] 3Kagami M, O'Sullivan MJ, Green AJ et al: The IG-DMR and the MEG3-DMR at human chromosome 14q32.2: hierarchical interaction and distinct functional properties as imprinting control centers. PLoS Genet 2010; 6: e1000992. 20585555 10.1371/journal.pgen.1000992 PMC2887472

[bib4] 4Beygo J, Elbracht M, de Groot K et al: Novel deletions affecting the MEG3-DMR provide further evidence for a hierarchical regulation of imprinting in 14q32. Eur J Hum Genet 2015; 23: 180–188. 24801763 10.1038/ejhg.2014.72 PMC4297900

[bib5] 5Hoffmann K, Heller R: Uniparental disomies 7 and 14. Best Pract Res Clin Endocrinol Metab 2011; 25: 77–100. 21396576 10.1016/j.beem.2010.09.004

[bib6] 6Kagami M, Matsuoka K, Nagai T et al: Paternal uniparental disomy 14 and related disorders: placental gene expression analyses and histological examinations. Epigenetics 2012; 7: 1142–1150. 22917972 10.4161/epi.21937 PMC3469456

[bib7] 7Kurosawa K, Sasaki H, Sato Y et al: Paternal UPD14 is responsible for a distinctive malformation complex. Am J Med Genet 2002; 110: 268–272. 12116236 10.1002/ajmg.10404

[bib8] 8Kagami M, Nishimura G, Okuyama T et al: Segmental and full paternal isodisomy for chromosome 14 in three patients: narrowing the critical region and implication for the clinical features. Am J Med Genet A 2005; 138A: 127–132. 16152632 10.1002/ajmg.a.30941

[bib9] 9Kagami M, Kato F, Matsubara K, Sato T, Nishimura G, Ogata T: Relative frequency of underlying genetic causes for the development of UPD(14)pat-like phenotype. Eur J Hum Genet 2012; 20: 928–932. 22353941 10.1038/ejhg.2012.26 PMC3421115

[bib10] 10Horii M, Horiuchi H, Momoeda M, Nakagawa M et al: Hepatoblastoma in an infant with paternal uniparental disomy 14. Congenit Anom (Kyoto) 2012; 52: 219–220. 23181499 10.1111/j.1741-4520.2012.00364.x

[bib11] 11Miyazaki O, Nishimura G, Kagami M, Ogata T: Radiological evaluation of dysmorphic thorax of paternal uniparental disomy 14. Pediatr Radiol 2011; 41: 1013–1019. 21607596 10.1007/s00247-011-2132-1

[bib12] 12Parveen Z, Tongson-Ignacio JE, Fraser CR, Killeen JL, Thompson KS: Placental mesenchymal dysplasia. Arch Pathol Lab Med 2007; 131: 131–137. 17227114 10.5858/2007-131-131-PMD

[bib13] 13Williams DH, Gauthier DW, Maizels M: Prenatal diagnosis of Beckwith–Wiedemann syndrome. Prenat Diagn 2005; 25: 879–884. 16193463 10.1002/pd.1155

[bib14] 14da Rocha ST, Tevendale M, Knowles E, Takada S, Watkins M, Ferguson-Smith AC: Restricted co-expression of Dlk1 and the reciprocally imprinted non-coding RNA, Gtl2: implications for cis-acting control. Dev Biol 2007; 306: 810–823. 17449025 10.1016/j.ydbio.2007.02.043

[bib15] 15da Rocha ST, Charalambous M, Lin SP et al: Gene dosage effects of the imprinted delta-like homologue 1 (dlk1/pref1) in development: implications for the evolution of imprinting. PLoS Genet 2009; 5: e1000392. 19247431 10.1371/journal.pgen.1000392 PMC2640098

[bib16] 16Sekita Y, Wagatsuma H, Nakamura K et al: Role of retrotransposon-derived imprinted gene, Rtl1, in the feto-maternal interface of mouse placenta. Nat Genet 2008; 40: 243–248. 18176565 10.1038/ng.2007.51

[bib17] 17Brandt J, Schrauth S, Veith AM et al: Transposable elements as a source of genetic innovation: expression and evolution of a family of retrotransposon-derived neogenes in mammals. Gene 2005; 345: 101–111. 15716091 10.1016/j.gene.2004.11.022

[bib18] 18Modena AB, Fieni S: Amniotic fluid dynamics. Acta Biomed 2004; 75: 11–13. 15301282

[bib19] 19Beall MH, van den Wijngaard JP, van Gemert MJ, Ross MG: Amniotic fluid water dynamics. Placenta 2007; 28: 816–823. 17254633 10.1016/j.placenta.2006.11.009

[bib20] 20Hurst LD, McVean GT: Growth effects of uniparental disomies and the conflict theory of genomic imprinting. Trends Genet 1997; 13: 436–443. 9385840 10.1016/s0168-9525(97)01273-0

[bib21] 21Georgiades P, Watkins M, Surani MA, Ferguson-Smith AC: Parental origin-specific developmental defects in mice with uniparental disomy for chromosome 12. Development 2000; 127: 4719–4728. 11023874 10.1242/dev.127.21.4719

[bib22] 22Wilkinson LS, Davies W, Isles AR: Genomic imprinting effects on brain development and function. Nat Rev Neurosci 2007; 8: 832–843. 17925812 10.1038/nrn2235

[bib23] 23Kang ER, Iqbal K, Tran DA et al: Effects of endocrine disruptors on imprinted gene expression in the mouse embryo. Epigenetics 2011; 6: 937–950. 21636974 10.4161/epi.6.7.16067 PMC3154434

[bib24] 24Riordan JD, Keng VW, Tschida BR et al: Identification of rtl1, a retrotransposon-derived imprinted gene, as a novel driver of hepatocarcinogenesis. PLoS Genet 2013; 9: e1003441. 23593033 10.1371/journal.pgen.1003441 PMC3616914

[bib25] 25Zhou Y, Zhang X, Klibanski A: MEG3 noncoding RNA: a tumor suppressor. J Mol Endocrinol 2012; 48: R45–R53. 22393162 10.1530/JME-12-0008 PMC3738193

[bib26] 26Floridon C, Jensen CH, Thorsen P et al: Does fetal antigen 1 (FA1) identify cells with regenerative, endocrine and neuroendocrine potentials? A study of FA1 in embryonic, fetal, and placental tissue and in maternal circulation. Differentiation 2000; 66: 49–59. 10997592 10.1046/j.1432-0436.2000.066001049.x

[bib27] 27Falix FA, Aronson DC, Lamers WH, Hiralall JK, Seppen J: DLK1, a serum marker for hepatoblastoma in young infants. Pediatr Blood Cancer 2012; 59: 743–745. 22180200 10.1002/pbc.24024

[bib28] 28Yamazawa K, Kagami M, Fukami M, Matsubara K, Ogata T: Monozygotic female twins discordant for Silver-Russell syndrome and hypomethylation of the H19-DMR. J Hum Genet 2008; 53: 950–955. 18709478 10.1007/s10038-008-0329-4

[bib29] 29Azzi S, Blaise A, Steunou V et al: Complex tissue-specific epigenotypes in Russell–Silver Syndrome associated with 11p15 ICR1 hypomethylation. Hum Mutat 2014; 35: 1211–1220. 25044976 10.1002/humu.22623

[bib30] 30Berend SA, Horwitz J, McCaskill C, Shaffer LG: Identification of uniparental disomy following prenatal detection of Robertsonian translocations and isochromosomes. Am J Hum Genet 2000; 66: 1787–1793. 10775524 10.1086/302916 PMC1378034

[bib31] 31Ioannides Y, Lokulo-Sodipe K, Mackay DJ, Davies JH, Temple IK: Temple syndrome: improving the recognition of an underdiagnosed chromosome 14 imprinting disorder: an analysis of 51 published cases. J Med Genet 2014; 51: 495–501. 24891339 10.1136/jmedgenet-2014-102396

[bib32] 32Hosoki K, Kagami M, Tanaka T et al: Maternal uniparental disomy 14 syndrome demonstrates Prader–Willi syndrome-like phenotype. J Pediatr 2009; 155: 900–903. 19800077 10.1016/j.jpeds.2009.06.045

[bib33] 33Wang JC, Passage MB, Yen PH, Shapiro LJ, Mohandas TK: Uniparental heterodisomy for chromosome 14 in a phenotypically abnormal familial balanced 13/14 Robertsonian translocation carrier. Am J Hum Genet 1991; 48: 1069–1074. 2035528 PMC1683099

[bib34] 34Kagami M, Yamazawa K, Matsubara K et al: Placentomegaly in paternal uniparental disomy for human chromosome 14. Placenta 2008; 29: 760–761. 18619672 10.1016/j.placenta.2008.06.001

