# Supplementary material for: Comprehensive clinical studies in 34 patients with molecularly defined UPD(14)pat and related conditions (Kagami–Ogata syndrome)
Source: Eur J Hum Genet. 2015 Feb 18;23(11):1488–98. doi: 10.1038/ejhg.2015.13 (PMC4613461; doi:10.1038/ejhg.2015.13)
Supplement: Supplementary Information [file ejhg201513x1.pdf]

Supplementary information

(Supplementary Tables 1–5, and Supplementary Figures 1–5)

Comprehensive clinical studies in 34 patients with molecularly defined UPD(14)pat and related conditions (Kagami-Ogata syndrome)

Masayo Kagami[1], Kenji Kurosawa[2], Osamu Miyazaki[3], Fumitoshi Ishino[4], Kentaro Matsuoka[5], Tsutomu Ogata[1,6]

[1]Department of Molecular Endocrinology, National Research Institute for Child Health and Development, Tokyo, Japan

[2]Division of Medical Genetics, Kanagawa Children's Medical Center, Yokohama, Japan

[3]Department of Radiology, National Center for Child Health and Development, Tokyo, Japan

[4]Department of Epigenetics, Medical Research Institute, Tokyo Medical and Dental University, Tokyo, Japan

[5]Department of Pathology, National Center for Child Health and Development, Tokyo, Japan

[6]Department of Pediatrics, Hamamatsu University School of Medicine, Hamamatsu, Japan

Table S1. A reference list of previously reported 33 Japanese, one Irish, and 17 other non-Japanese patients with UPD(14)pat and related conditions (Kagami-Ogata syndrome)

References	Patient No. in this report
1 Wang JC, Passage MB, Yen PH, Shapiro LJ, Mohandas TK: Uniparental heterodisomy for chromosome 14 in a phenotypically abnormal familial balanced 13/14 Robertsonian translocation carrier. *Am J Hum Genet* 1991; **48:** 1069-1074.	#35
2 Papenhausen PR, Mueller OT, Johnson VP, Sutcliffe M, Diamond TM, Kousseff BG: Uniparental isodisomy of chromosome 14 in two cases: an abnormal child and a normal adult. *Am J Med Genet*1995; **59:** 271-275.	#36
3 Walter CA, Shaffer LG, Kaye CI *et al*: Short-limb dwarfism and hypertrophic cardiomyopathy in a patient with paternal isodisomy 14: 45,XY,idic(14)(p11). *Am J Med Genet* 1996; **65:** 259-265.	#37
4 Cotter PD, Kaffe S, McCurdy LD, Jhaveri M, Willner JP, Hirschhorn K: Paternal uniparental disomy for chromosome 14: a case report and review. *Am J Med Genet* 1997; **70:** 74-79.	#38
5 Klein J, Shaffer L, McCaskill C *et al*: Delineation of the paternal disomy 14 syndrome: Identification of a case by prenatal diagnosis. *Am J Hum Genet* 1999; **65 (Supple):** A179.	#39
6 Yano S, Li L, Owen S, Wu S, Tran T: A further delineation of the uniparental disomy (UPD 14): The fifth reported liveborn case. *Am J Hum Genet* 2001; **69 (Supple):** A739.	#40
7 Kurosawa K, Sasaki H, Sato Y *et al*: Paternal UPD14 is responsible for a distinctive malformation complex. *Am J Med Genet* 2002; **110**: 268-272.	#12, #17, #23
8 Coveler KJ, Yang SP, Sutton R *et al*: A case of segmental paternal isodisomy of chromosome 14. *Hum Genet* 2002; **110:** 251-256.	#41
9 McGowan KD, Weiser JJ, Horwitz J *et al*: The importance of investigating for uniparental disomy in prenatally identified balanced acrocentric rearrangements. *Prenat Diagn* 2002; **22:** 141-143.	#42
10 Offiah AC, Cornette L, Hall CM: Paternal uniparental disomy 14: introducing the "coat-hanger" sign. *Pediatr Radiol* 2003; **33:** 509-512.	#43
11 Chu C, Schwartz S, McPherson E: Paternal uniparental isodisomy for chromosome 14 in a patient with a normal 46,XY karyotype. *Am J Med Genet A* 2004; **127A:** 167-171.	#44
12 Stevenson DA, Brothman AR, Chen Z, Bayrak-Toydemir P, Longo N: Paternal uniparental disomy of chromosome 14: confirmation of a clinically-recognizable phenotype. *Am J Med Genet A* 2004; **130A:** 88-91.	#45
13 Kagami M, Nishimura G, Okuyama T *et al:* Segmental and full paternal isodisomy for chromosome 14 in three patients: narrowing the critical region and implication for the clinical features. *Am J Med Genet A* 2005; **138A**: 127-132.	#1, #11, #14
14 Curtis L, Antonelli E, Vial Y *et al*: Prenatal diagnostic indicators of paternal uniparental disomy 14. *Prenat Diagn* 2006; **26:** 662-666.	#46

15	Mattes J, Whitehead B, Liehr T *et al*: Paternal uniparental isodisomy for chromosome 14 with mosaicism for a supernumerary marker chromosome 14. *Am J Med Genet A* 2007; **143A:** 2165-2171.	#47
16	Kagami M, Sekita Y, Nishimura G *et al*: Deletions and epimutations affecting the human 14q32.2 imprinted region in individuals with paternal and maternal upd(14)-like phenotypes. *Nat Genet* 2008; **40:** 237-242.	#24, #27, #28, #30, #31, #33, #34
17	Kagami M, O'Sullivan MJ, Green AJ *et al*: The IG-DMR and the MEG3-DMR at human chromosome 14q32.2: hierarchical interaction and distinct functional properties as imprinting control centers. *PLoS Genet* 2010; **6:** e1000992.	#29, #32
18	Irving MD, Buiting K, Kanber D *et al*: Segmental paternal uniparental disomy (patUPD) of 14q32 with abnormal methylation elicits the characteristic features of complete patUPD14. *Am J Med Genet A* 2010; **152A:** 1942-1950.	#48
19	Horii M, Horiuchi H, Momoeda M, Nakagawa M *et al*: Hepatoblastoma in an infant with paternal uniparental disomy 14. *Congenit Anom (Kyoto)* 2012; **52:** 219-220.	#18
20	Kagami M, Kato F, Matsubara K, Sato T, Nishimura G, Ogata T: Relative frequency of underlying genetic causes for the development of UPD(14)pat-like phenotype. *Eur J Hum Genet* 2012; **20:** 928-932.	#3 ~ #10, #13, #15, #16, #21, #22, #26,
21	Beygo J, Elbracht M, de Groot K *et al*: Novel deletions affecting the MEG3-DMR provide further evidence for a hierarchical regulation of imprinting in 14q32. *Eur J Hum Genet* [Epub ahead of print].	#49 ~ #51
22	This report	#2, #19, #20, #25,

Table S2. Clinical information of each patient examined in this study

	Subtype 1 Sporadic Patient 1	Subtype 1 Sporadic Patient 2	Subtype 1 Sporadic Patient 3	Subtype 1 Sporadic Patient 4	Subtype 1 Sporadic Patient 5	Subtype 1 Sporadic Patient 6	Subtype 1 Sporadic Patient 7	Subtype 1 Sporadic Patient 8	Subtype 1 Sporadic Patient 9
Age at the last examination or death (y)	2 hours	1	1 1/12	2 4/12	2 11/12	3	3	3 2/12	4 4/12
Sex (male:female)	Male	Male	Female	Female	Female	Male	Male	Male	Female
Karyotype	N.E.	46,XY	46,XX	46,XX	46,XX	46,XY	46,XY	46,XY	46,XX
<Molecular findings>									
IG-DMR of maternal origin	Absent	Absent	Absent	Absent	Absent	Absent	Absent	Absent	Absent
MEG3 -DMR of maternal origin	Absent	Absent	Absent	Absent	Absent	Absent	Absent	Absent	Absent
DLK1 expression level	2 ×	2 ×	2 ×	2 ×	2 ×	2 ×	2 ×	2 ×	2 ×
RTL1 expression level	~5 ×	~5 ×	~5 ×	~5 ×	~5 ×	~5 ×	~5 ×	~5 ×	~5 ×
MEGs expression level	0 ×	0 ×	0 ×	0 ×	0 ×	0 ×	0 ×	0 ×	0 ×
Generation of UPD(14)pat	MR/PE	MR/PE	MR/PE	MR/PE	MR/PE	MR/PE	MR/PE	MR/PE	MR/PE
<Pregnancy and delivery>									
Polyhydramnios	+	+	+	+	+	+	+	Probable	+
Gestational age at Dx (w)	Unknown	24	Unknown	19	22	30	26[m]	Unknown	30
Amnioreduction (w)	Unknown	6x (25–31)	1x (33)	6x (23–34)	1x (29)	2x (33,36)	4x (26–35)	None	3x (Unknown)
Placentomegaly[a]	–	+	Unknown	+	Unknown	–	+	–	+
Placental weight g (%)	556 (114)	670 (137)	Unknown	1110 (227)	Unknown	570 (117)	1384 (262)	278 (114)	1108 (227)
Prenatal Dx of thoracic abnormality	Unknown	–	–	–	+	–	+	–	–
Gestational age at Dx (w)	Unknown	…	…	…	22	…	28[m]	…	…
Prenatal Dx of abdominal abnormality	Unknown	+	–	+	–	–	+	–	–
Gestational age at Dx (w)	Unknown	Unknown	…	22	…	…	28[m]	…	…
Gestational age (w)	34	33	34	35	35	36	37	24	34
Premature delivery (<37 w)	+	+	+	+	+	+	–	+	+
Delivery	Caesarean	Caesarean	Caesarean	Caesarean	Caesarean	Vaginal	Caesarean	Caesarean	Caesarean
Record of conception	–	+	+	+	+	+	+	+	+
Medically assisted reproduction	Unknown	–	–	–	–	–	–	+ (IVF–ET)	–
<Growth deficiency>									
Prenatal growth failure[b]	–	–	–	–	–	–	–	–	–
Prenatal overgrowth[c]	+	+	–	+	+	–	–	+	+
Birth length cm (SD)	45.7 (+0.7)	Unknown	42.5 (–0.7)	48 (+1.4)	51 (+3.0)	46.2 (±0)	48 (+0.4)	30.6 (+0.3)	44 (±0)
Birth weight kg (SD)	3.17 (+4.2)	2.55 (+2.7)	2.05 (+0.2)	2.92 (+2.4)	2.67 (+1.6)	2.85 (+1.3)	2.70 (+0.2)	1.24 (+8.8)	2.79 (+2.7)
Postnatal growth failure[d]	…	+	–	+	–	+	–	+	–
Postnatal overgrowth[e]	…	–	–	–	–	–	–	–	–
Present stature cm (SD)	…	59 (–6.2)	76.0 (+0.8)	77.8 (–3.0)	90.5 (–0.3)	70 (–6.4)	90 (–0.9)	61.8 (–8.7)	99.3 (–0.6)
Present weight kg (SD)	…	5.2 (–4.6)	9.5 (–0.5)	10.1 (–1.3)	14.9 (+1.4)	8.3 (–3.4)	12.8 (–0.6)	4.2 (–6.0)	14.2 (–1.0)
<Craniofacial and cervical features>									
Frontal bossing	–	+	+	+	+	+	+	+	+
Hairy forehead	+	±	–	+	+	+	+	+	–
Blepharophimosis	–	+	+	–	+	+	+	+	+
Small ears	+	+	+	–	–	+	–	–	–
Depressed nasal bridge	+	+	+	+	+	+	+	+	+
Anteverted nares	+	+	+	–	+	+	+	+	+
Full cheeks	Unknown	+	+	+	+	+	+	+	+
Protruding philtrum	+	+	+	+	+	+	+	+	+
Puckered lips	–	+	–	+	+	±	+	–	+
Micrognathia	+	+	+	+	+	+	+	+	+
Short webbed neck	+	+	+	+	+	+	+	+	+
<Thoracic abnormality>									
Small bell-shaped thorax in infancy[f]	+	+	+	+	+	+	+	+	+
Coat-hanger appearance in infancy[g]	+	+	+	+	+	+	+	+	+
Laryngomalacia	…	–	–	–	–	Unknown	+	+	–
Tracheostomy	…	+	–	+	–	+	–	+ (LTS)	–
Mechanical ventilation (duration)[h]	+ (2 hrs)	+ (On ventilation)	+ (5 months)	+ (12 months)	+ (2 days)	+ (On ventilation)	+ (6 days)	+ (9 months)	+ (65 days)
Oxygen administration (duration)[i]	+ (2 hrs)	+ (Ongoing)	+ (20 days)	+ (3 months)	+ (2 days)	+ (Ongoing)	+ (1 day)	+ (34 months)	+ (7 months)
Respiratory rehabilitation	…	+	–	–	–	–	–	+	–
<Abdominal wall defect>	+	+	+	+	+	+	+	+	+
Omphalocele	+	+	+	+	+	–	–	–	–
Diastasis recti	–	–	–	–	–	+	+	+	+
Constipation (treatment)	…	+ (enema)	–	–	+ (enema)	–	+ (laxatives)	+ (enema)	–
<Development>									
Developmental delay	…	+	+	+	+	+	+	+	+
DQ/IQ (age at examination)	…	N.E.	58 (1y)	N.E.	55 (2y8m)	N.E.	70 (Unknown)	N.E.	64 (3y4m)
Age at head control (m)	…	12	7	7	5	36	7	7	6
Age at sitting without support (m)	…	–	–	10	Unknown	–	12	–	12
Age at walking without support (m)	…	…	…	–	24	–	22	–	30
Education	…	…	…	…	…	…	…	…	…
<Other features>									
Feeding difficulty	…	+	+	+	+	+	+	+	+
Tube feeding (duration)[j]	…	+ (Ongoing)	+ (Ongoing)	+ (Ongoing)	+ (2 days)	+ (Ongoing)	+ (6 months)	+ (Unknown)	+ (6 months)
Rehabilitation for eating	…	–	+	+	–	–	–	Unknown	+
Joint contractures	–	+	–	–	+	–	+	+	+
Kyphoscoliosis	…	–	+	–	–	+	–	–	+
Coxa valga	…	+	–	–	–	±	–	±	+
Cardiac disease	+ (ASD)	–	–	–	+ (small ASD)	+ (IAA & VSD)	–	+ (PDA)	–
Inguinal hernia	–	+	–	–	–	–	–	+	–
Seizure	…	–	–	–	–	–	–	–	–
Hepatoblastoma (age at diagnosis)	–	–	–	–	–	–	–	+ (13 months)	–
<Mortality>									
Alive:Deceased	Deceased	Alive	Alive	Alive	Alive	Alive	Alive	Deceased	Alive
Causes of death	Respiratory failure	…	…	…	…	…	…	Influenza infection	…
<Parents>									
Paternal age at childbirth (y)	Unknown	35	28	34	38	33	36	37	28
Maternal age at childbirth (y)	42	33	30	39	38	32	41	42	31
Extra features	Unknown	Milk allergy	–	–	–	–	–	–	–
LOVD Individual ID[k]	00027235	00027236	00027237	00027238	00027239	00027240	00027241	00027242	00027243
ClinVer SCV accession number[l]	…	…	…	…	…	…	…	…	…
References	2	This report	3	3	3	3	3	3	3

UPD(14)pat									
Subtype 1	Subtype 1	Subtype 1	Subtype 1	Subtype 2	Subtype 3	Subtype 3	Subtype 3	Subtype 3	Subtype 3
Sporadic	Sporadic	Sporadic	Sporadic	Sporadic	Sporadic	Sporadic	Sporadic	Sporadic	Sporadic
Patient 10	Patient 11	Patient 12	Patient 13	Patient 14	Patient 15	Patient 16	Patient 17	Patient 18	Patient 19
5 7/12	7 2/12	11 11/12	15	12	6/12	6/12	8/12	1 5/12	1 7/12
Female	Female	Male	Male	Female	Male	Male	Female	Female	Female
46,XX	46,XX	46,XY	46,XY	46,XX	46,XY	46,XY	45,XX,rob(13;14)(q10;q10)[n]	46,XX	46,XX
Absent	Absent	Absent	Absent	Absent	Absent	Absent	Absent	Absent	Absent
Absent	Absent	Absent	Absent	Absent	Absent	Absent	Absent	Absent	Absent
2 ×	2 ×	2 ×	2 ×	2 ×	2 ×	2 ×	2 ×	2 ×	2 ×
~5 ×	~5 ×	~5 ×	~5 ×	~5 ×	~5 ×	~5 ×	~5 ×	~5 ×	~5 ×
0 ×	0 ×	0 ×	0 ×	0 ×	0 ×	0 ×	0 ×	0 ×	0 ×
MR/PE	MR/PE	MR/PE	MR/PE	PE	TR/GC	TR/GC	TR/GC	TR/GC	TR/GC
+	+	+	+	+	+	+	+	+	+
Unknown	24	Unknown	19	28	26	14	26	24	22
3x (22–34)	3x (29–32)	Unknown	3x (27–32)	2x (29,34)	4x (26–32)	7x (32–37)	Unknown	6x (30–37)	None
+	+	Unknown	+	+	+	+	Unknown	+	+
750 (142)	635 (161)	Unknown	930 (190)	1108 (227)	640 (131)	1030 (195)	Unknown	990 (187)	430 (143)
–	–	Unknown	–	–	+	Unknown	+	+	+
…	…	Unknown	…	…	26	Unknown	26	33	23
–	–	Unknown	–	–	+	Unknown	Unknown	Unknown	–
…	…	Unknown	…	…	26	Unknown	Unknown	Unknown	…
37	32	33	35	36	34	37	32	37	28
–	+	+	+	+	+	–	+	–	+
Caesarean	Caesarean	Caesarean	Caesarean	Vaginal	Vaginal	Caesarean	Vaginal	Vaginal	Vaginal
+	–	–	+	+	+	–	–	+	+
–	Unknown	Unknown	–	–	–	Unknown	Unknown	–	–
–	–	–	–	–	–	–	–	–	–
–	–	+	+	–	–	–	–	–	+
44.5 (–1.2)	41 (–0.4)	45 (+0.9)	45 (–0.1)	46 (–0.1)	47 (+1.4)	49 (+0.9)	43 (+0.5)	43.5 (–1.7)	36 (–0.2)
2.80 (+0.8)	1.85 (+0.6)	2.80 (+3.1)	3.14 (+3.1)	2.94 (+1.8)	2.50 (+1.6)	3.10 (+1.5)	1.85 (+0.8)	2.56 (+0.1)	1.61 (+3.2)
–	–	–	–	–	Unknown	+	Unknown	+	+
–	–	–	–	–	Unknown	–	Unknown	–	–
103 (–1.6)	110.6 (–1.8)	148.3 (±0)	159.0 (–1.1)	133.4 (+1.1) at 8y 6m	Unknown	61.5 (–2.6)	Unknown	71.8 (–2.4)	75.8 (–1.6)
18.6 (±0)	17.7 (–1.3)	39.5 (–0.2)	38.0 (–1.8)	33.6 (+1.2) at 8y 6m	Unknown	7.1 (–0.9)	Unknown	8.3 (–1.4)	8.5 (–1.5)
+	+	Unknown	+	–	–	+	+	+	–
+	+	Unknown	+	+	+	+	+	+	–
+	+	Unknown	+	+	+	+	+	–	+
+	±	Unknown	–	–	–	–	Unknown	+	–
+	+	+	+	+	+	+	+	+	+
+	+	Unknown	+	–	+	+	+	–	+
+	+	Unknown	+	+	+	+	–	+	+
+	+	+	+	+	+	+	+	+	+
–	–	Unknown	–	–	+	+	Unknown	+	+
+	+	Unknown	+	+	+	+	+	–	+
+	+	+	+	+	+	+	Unknown	+	+
+	+	+	+	+	+	+	+	+	+
+	+	+	+	+	+	+	+	+	+
–	–	+	–	+	–	±	Unknown	–	+
–	–	–	–	–	–	+	Unknown	+	–
+ (40 days)	+ (31 days)	+ (15days)	+ (2 days)	–	+ (6 months)	+ (On ventilation)	+ (Unknown)	+ (17 months)	+ (2 months)
–	+ (52 months)	+ (18 months)	+ (5 days)	+ (12 months)	+ (Unknown)	+ (Ongoing)	+ (Unknown)	+ (27 days)	+ (17 months)
–	–	–	–	–	–	Unknown	Unknown	–	–
+	+	+	+	+	+	+	+	+	+
–	–	–	–	–	–	–	–	–	–
+	+	+	+	+	+	+	+	+	+
–	(enema, laxative	+ (enema)	–	+ (laxatives)	+	Unknown	Unknown	….	+ (enema, laxatives)
+	+	+	+	+	+	+	Unknown	+	+
59 (3y3m)	50 (6y3m)	29 (7y3m)	65 (Unknown)	46 (9y1m)	N.E.	N.E.	N.E.	N.E.	47 (1y7m)
Unknown	3	Unknown	10	4	Unknown	Unknown	Unknown	9	9
19	9	Unknown	12	10	…	Unknown	…	13	13
33	25	26	24	23	…	…	…	–	–
…	Regular class	Special class	Special class	Regular class	…	…	…	….	….
+	+	+	+	+	+	+	Unknown	+	+
+ (36 months)	+ (9 months)	+ (Unknown)	+ (1 month)	+ (18 days)	+ (6 months)	+ (Ongoing)	Unknown	+ (17 months)	+ (Ongoing)
+	+	Unknown	–	–	–	Unknown	Unknown	–	+
+	+	–	+	+	+	–	Unknown	–	+
–	+	–	–	+	+	–	Unknown	+	–
–	+	–	–	–	–	–	Unknown	+	–
–	–	–	–	–	–	–	Unknown	–	+ (PDA)
–	–	+	+	–	–	–	Unknown	–	+
+	–	–	–	–	–	–	Unknown	–	–
–	–	–	–	–	–	–	+ (46 days)	+ (218 days)	–
Alive	Alive	Alive	Alive	Alive	Deceased	Alive	Deceased	Deceased	Alive
…	…	…	…	…	NEC	…	Hepatoblastoma	HPS	…
35	36	26	32	24	35	41	31	36	36
26	36	32	26	27	31	36	28	29	28
–	–	Cryptorchidism	–	–	NEC	–	Unknown	HPS	–
00027244	00027245	00027246	00027247	00027248	00027250	00027251	00027252	00027253	00027254
…	…	…	…	…	…	…	…	…	…
3	2	4	3	2	3	3	4	5	This report

				Epimutations				
Subtype 3	Subtype 3	Subtype 3	Subtype 3					
Sporadic	Sporadic	Sporadic	Sporadic	Sporadic	Sporadic	Sporadic	Sporadic	Sporadic
Patient 20	Patient 21	Patient 22	Patient 23	Patient 24	Patient 25	Patient 26	Patient 27	Patient 28
1 8/12	2 3/12	7	9 8/12	9/12	10/12	2	4 7/12	5 6/12
Female	Female	Female	Female	Female	Male	Male	Female	Male
45,XX,rob(14;21)(q10;q10)[n]	46,XX	46,XX	46,XX	46,XX	46,XY	46,XY	46,XX	46,XY
Absent	Absent	Absent	Absent	Methylated	Methylated	Methylated	Methylated	Methylated
Absent	Absent	Absent	Absent	Methylated[o]	Methylated[o]	Methylated[o]	Methylated[o]	Methylated[o]
2 ×	2 ×	2 ×	2 ×	2 ×	2 ×	2 ×	2 ×	2 ×
~5 ×	~5 ×	~5 ×	~5 ×	~5 ×	~5 ×	~5 ×	~5 ×	~5 ×
0 ×	0 ×	0 ×	0 ×	0 ×	0 ×	0 ×	0 ×	0 ×
TR/GC	TR/GC	TR/GC	TR/GC	…	…	…	…	…
+	+	+	+	+	+	+	+	+
Unknown	22[m]	26	25	Unknown	26	30	22	29
4x (30–35)	3x (22–34)	5x (25–35)	2x (25, 29)	2x (27–29)	2x (30, 31)	None	4x (29–34)	4x (29–34)
+	+	Unknown	Unknown	+	+	Unknown	+	+
930 (176)	970 (198)	Unknown	Unknown	615 (156)	1070 (218)	Unknown	710 (145)	680 (139)
–	+	–	+	Unknown	+	–	+	Unknown
…	33[m]	…	Unknown	Unknown	30	…	25	Unknown
–	+	–	+	Unknown	+	+	+	Unknown
…	Unknown	…	Unknown	Unknown	Unknown	Unknown	25	Unknown
38	36	37	32	30	35	37	35	35
–	+	–	+	+	+	–	+	+
Vaginal	Caesarean	Vaginal	Caesarean	Caesarean	Caesarean	Caesarean	Vaginal	Caesarean
+	+	+	+	–	+	–	–	–
–	–	–	–	Unknown	–	Unknown	Unknown	Unknown
–	–	–	–	–	–	–	–	–
+	+	+	+	–	–	–	+	+
50 (+1.0)	45 (–0.5)	48.5 (+0.6)	Unknown	41 (+0.9)	44 (–0.5)	50 (+1.4)	43.5 (–0.7)	43 (–0.9)
3.77 (+3.2)	3.40 (+3.0)	3.50 (+2.9)	2.21 (+2.2)	1.61 (+1.2)	3.28 (+3.7)	2.80 (+0.5)	2.93 (+2.5)	2.90 (+2.2)
–	–	–	+	+	–	–	+	–
+	–	–	–	–	–	+	–	–
85.5 (–1.5)	78.6 (–1.9)	122.3 (+0.6)	101.2 (–5.0) at 9y0m	54.0 (–7.1)	68.4 (–1.8)	85.0 (–0.1)	88.5 (–2.2) at 3y7m	112.9 (+0.9)
13.0 (+2.4)	10.1 (–1.0)	23.9 (+0.4)	17.9(–1.9) at 9y0m	3.4 (–5.5)	8.1 (–1.1)	17.0 (+4.0)	13.5 (–0.6) at 3y7m	20.8 (+1.0)
+	+	–	+	±	+	+	+	–
+	+	–	+	+	–	–	–	–
+	+	–	+	+	–	+	+	–
+	–	–	–	–	+	–	–	+
+	+	+	+	+	+	+	+	+
+	+	+	+	–	+	+	±	+
+	+	+	+	Unknown	+	+	+	+
+	+	+	+	+	+	+	+	+
–	–	–	+	–	+	+	–	+
Unknown	+	+	+	+	+	+	+	+
+	+	+	+	+	+	+	+	+
+	+	+	+	+	+	+	+	+
+	+	+	+	+	+	+	+	+
+	–	–	+	–	+	–	–	+
–	–	–	+	Unknown	–	–	+	–
– (n–CPAP)	+ (5 days)	+ (9 days)	+ (17 months)	+ (On ventilation)	+ (21 days)	+ (3 days)	+ (On ventilation)	+ (27 days)
+ (Unknown)	+ (20 months)	+ (2 days)	+ (50 months)	Unknown	+ (27 days)	+ (3 days)	+ (On going)	+ (6 months)
–	+	–	+	Unknown	–	–	+	–
+	+	+	+	+	+	+	+	+
–	+	–	+	+	–	+	–	–
+	–	+	–	–	+	–	+	+
+ (enema)	+	+ (laxatives)	+ (enema)	Unknown	–	+ (enema)	+ (enema)	+ (enema, laxatives)
+	+	+	+	+	+	+	+	+
N.E.	42 (Unknown)	60 (2y8m)	32 (7y1m)	N.E.	N.E.	N.E.	48 (3y10m)	56 (5y6m)
Unknown	10	6	14	Unknown	8	6	11	6
Unknown	17	8	25	Unknown	10	12	20	11
20	27	27	49	…	…	22	39	25
….	….	Regular class	Special class	…	…	…	…	…
+	+	–	+	+	+	+	+	+
+ (6 months)	+ (Ongoing)	–	+ (72 months)	+ (Unknown)	+ (30 days)	+ (14 days)	+ (17 months)	+ (16 months)
+	+	–	+	Unknown	+	–	+	+
+	–	+	+	+	–	+	–	+
–	–	+	+	+	+	–	+	–
–	–	–	–	–	–	–	–	±
–	–	–	–	–	PS	–	–	–
–	–	–	–	+	–	–	–	–
–	–	–	–	–	–	–	–	–
–	–	–	–	–	–	–	–	–
Alive	Alive	Alive	Alive	Alive	Alive	Alive	Alive	Alive
…	…	…	…	…	…	…	…	…
47	29	38	26	26	27	33	30	36
43	28	29	25	28	25	33	27	35
–	–	Hepatosplenomegaly	–	–	–	–	–	–
00027255	00027256	00027257	00027258	00027230	00027231	00027232	00027233	00027234
…	…	…	…	…	…	…	…	…
This report	3	3	4	6	This report	3	6	6

Microdeletions					
Subtype 1	Subtype 1	Subtype 1	Subtype 2	Subtype 3	Subtype 3
Familial	Familial	Familial	Sporadic	Sporadic	Familial
Patient 29	Patient 30	Patient 31	Patient 32[p]	Patient 33	Patient 34
2 9/12	9/12	8 11/12	4 days	3 10/12	5 1/12
Female	Female	Male	Female	Female	Female
46,XX	46,XX	46,XY	46,XX	46,XX	46,XX
Deleted	Deleted	Deleted	Unmethylated	Deleted	Deleted
Methylated[o]	Deleted	Deleted	Deleted	Deleted	Deleted
2 ×	1 ×	1 ×	2 × (1 ×)[q]	2 ×	1×
~5 ×	~5 ×	~5 ×	~5 × (1 × or ~2.5 ×)[q]	~2.5 ×	~2.5 ×
0 ×	0 ×	0 ×	0 × (1 × or 0 ×)[q]	0 ×	0 ×
…	…	…	…	…	…
+	+	+	–	+	+
Unknown	Unknown	Unknown	…	Unknown	21
2x (28, 30)	None	5x (27–30)	…	3x (34–35)	None
+	+	+	–	+	+
850 (174)	429 (143)	642 (163)	…	732 (152)	600 (147)
–	Unknown	Unknown	Unknown	Unknown	–
…	Unknown	Unknown	Unknown	Unknown	…
+	Unknown	Unknown	Unknown	Unknown	–
Unknown	Unknown	Unknown	Unknown	Unknown	…
33	27	30	28	35	30
+	+	+	+	+	+
Caesarean	Vaginal	Caesarean	Vaginal	Caesarean	Caesarean
+	–	–	–	–	+
–	Unknown	Unknown	Unknown	Unknown	–
–	–	–	–	–	–
+	+	+	–	+	–
43 (±0)	Unknown	Unknown	34 (–1.1)	45 (–0.1)	42 (+1.5)
2.84 (+3.7)	1.30 (+2.4)	2.04 (+2.8)	1.32 (+1.5)	2.94 (+2.5)	1.55 (+0.9)
–	+	+	…	–	–
–	–	–	…	–	–
86 (–1.3)	59.5 (–2.2)	112 (–3.3)	…	Unknown	105 (–1.6)
12.3 (±0)	6.2 (–2.2)	21.4 (–1.3)	…	12.4 (–1.3)	16.8 (–0.9)
–	–	±	+	±	+
+	+	+	+	–	–
+	+	–	–	–	+
+	–	–	+	– (deformed)	–
+	+	+	–	–	+
+	+	+	–	±	+
+	Unknown	±	–	Unknown	+
+	+	+	–	±	+
+	+	+	–	–	–
+	+	+	+	–	+
+	+	+	+	+	+
+	+	+	+	+	+
+	+	+	+	+	+
–	+	+	…	–	Unknown
–	Unknown	–	…	+	+
+ (7 days)	+ (5 months)	+ (10 months)	+ (4 days)	+ (2 months)	+ (1 months)
+ (12 months)	Unknown	+ (103 months)	Unknown	+ (Unknown)	+ (36 months)
Unknown	Unknown	Unknown	Unknown	Unknown	–
+	+	+	+	+	+
+	–	–	+	–	–
–	+	+	–	±	±
–	Unknown	+ (enema)	…	–	–
+	+	+	…	+	+
N.E.	N.E.	N.E.	…	N.E.	N.E.
6	Unknown	Unknown	…	Unknown	6
18	Unknown	27	…	Unknown	18
30	…	90	…	–	24
…	…	Special class	…	…	…
+	+	+	…	+	+
+ (30 months)	+ (Unknown)	+ (89 months)	…	+ (46 months)	+ (51 months)
–	Unknown	+	…	Unknown	–
+	+	+	–	–	–
–	Unknown	+	–	–	Uncertain
+	±	+	–	+	–
–	–	–	+ (PDA)	–	+ (PDA)
–	+	+	–	–	–
–	–	–	–	–	–
–	–	–	–	–	–
Alive	Deceased	Alive	Deceased	Deceased	Alive
…	Sudden death at URI	…	Intracranial hemorrhage	RS virus infection	…
37	34	39	25	36	27
27	31	36	25	33	28
–	–	–	Hydronephrosis	UC, Asthma	–
28648	00027193	00027218	28649	00027215	00027213
SCV000190041	SCV000190038	SCV000190038	SCV000190042	SCV000190039	SCV000190040
7	6	6	7	6	6

Methylation patterns of the IG-DMR and the *MEG3*-DMR, and expression patterns of the imprinted genes, are illustrated in Supplementary Figure S1.
DQ/IQ: developmental/intellectual quotient; y: year; m: month; w: week; Dx: diagnosis; SD: standard deviation; MR: monosomy rescue; PE: postzygotic mitotic error; TR: trisomy rescue; GC: gamete complementation; N.E.: not examined; IVF-ET: in vitro fertilization and embryo transfer; LTS: laryngotracheal separation; n-CPAP: nasal continuous positive airway pressure; ASD: atrial septal defect; IAA: interrupted aortic arch; VSD: ventricular septal defect; PDA: patent ducts arteriosus; PS: pulmonary artery stenosis; URI: upper respiratory infection; NEC: necrotizing enterocolitis; HPS: hemophagocytic syndrome; RS: respiratory syncytial; and UC: ulcerative colitis.
[a] Placental weight >120% evaluated by the Japanese placental weight data reported by Kagami et al.[1]
[b] Birth length and/or birth weight < – 2 SD of the gestational age- and sex-matched Japanese reference data (http://jspe.umin.jp/medical/keisan.html).
[c] Birth length and/or birth weight > +2 SD of the gestational age- and sex-matched Japanese reference data (http://jspe.umin.jp/medical/keisan.html).
[d] Present length/height and/or present weight < – 2 SD of the age- and sex-matched Japanese reference data (http://jspe.umin.jp/medical/taikaku.html).
[e] Present length/height and/or present weight > +2 SD of the age- and sex-matched Japanese reference data (http://jspe.umin.jp/medical/taikaku.html).
[f] The ratio of mid to widest thorax diameter (M/W ratio) below normal range (see Figure 2).
[g] The coat-hanger angle (CHA) to the ribs above the normal range (see Figure 2).
[h] Duration in patients in whom mechanical ventilation could be discontinued.
[i] Duration in patients in whom oxygen administration could be discontinued.
[j] Duration in patients in whom tube feeding could be discontinued.
[k] Individual ID in the Leiden Open Variation Database (LOVD) (http://www.lovd.nl/3.0/home). Clinical information of each patient is seen at http://databases.lovd.nl/shared/individuals.
[l] Accession number in the the ClinVar database (http://www.ncbi.nlm.nih.gov/clinvar/).
[m] Diagnosis of UPD(14)pat has been suspected.
[n] The parental karyotype was not examined in patient #17 and #20 with Robertsonian translocations because of parental refusal, so it is unknown whether the Robertsonian translocations are inherited or produced by a *de novo* event.
[o] The *MEG3*-DMR is predicted to be grossly hypomethylated in the placenta.[7]
[p] Patient #32 is Irish, and the remaining patients are Japanese; the Irish patient has also been examined by Beygo *et al*.[8]
[q] Expression dosage of *RTL1* is predicted to be different between the body and the placenta in patient #32,[7] whereas it is predicted to be identical between the body and the placenta in other patients.

References

1. Kagami M, Yamazawa K, Matsubara K *et al*: Placentomegaly in paternal uniparental disomy for human chromosome 14. *Placenta* 2008; **29:** 760-761.
2. Kagami M, Nishimura G, Okuyama T *et al*: Segmental and full paternal isodisomy for chromosome 14 in three patients: narrowing the critical region and implication for the clinical features. *Am J Med Genet A* 2005; **138A:** 127-132.
3. Kagami M, Kato F, Matsubara K *et al*: Relative frequency of underlying genetic causes for the development of UPD(14)pat-like phenotype. *Eur J Hum Genet* 2012; **20:** 928-932.
4. Kurosawa K, Sasaki H, Sato Y *et al*: Paternal UPD14 is responsible for a distinctive malformation complex. *Am J Med Genet* 2002; **110:** 268-272.
5. Horii M, Horiuchi H, Momoeda M, Nakagawa M *et al*: Hepatoblastoma in an infant with paternal uniparental disomy 14. *Congenit Anom (Kyoto)* 2012; **52:** 219-220.
6. Kagami M, Sekita Y, Nishimura G *et al*: Deletions and epimutations affecting the human 14q32.2 imprinted region in individuals with paternal and maternal upd(14)-like phenotypes. *Nat Genet* 2008; **40:** 237-242.
7. Kagami M, O'Sullivan MJ, Green AJ *et al*: The IG-DMR and the MEG3-DMR at human chromosome 14q32.2: hierarchical interaction and distinct functional properties as imprinting control centers. *PLoS Genet* 2010; **6:** e1000992.
8. Beygo J, Elbracht M, de Groot K *et al*: Novel deletions affecting the MEG3-DMR provide further evidence for a hierarchical regulation of imprinting in 14q32. *Eur J Hum Genet* [Epub ahead of print].

Table S3. Clinical manifestations in 23 Japanese patients with different subtypes of UPD(14)pat

	UPD(14)pat			
	Subtype 1<br>MR/PE<br>Pts 1–13<br>(n=13)	Subtype 2<br>PE<br>Pt 14<br>(n=1)	Subtype 3<br>TR/GC<br>Pts 15–23<br>(n=9)	Subtotal<br><br>Pts 1–23<br>(n=23)
Age at the last examination or death (y)	3.0 (0.0–15.0)	12	1.6 (0.5–9.7)	2.9 (0.0–15.0)
Sex (male:female)	7:6	0:1	2:7	9:14
<Molecular findings[a]>				
IG-DMR of maternal origin	Absent	Absent	Absent	
MEG3 -DMR of maternal origin	Absent	Absent	Absent	
DLK1 expression level	2 ×	2 ×	2 ×	
RTL1 expression level	~5 ×	~5 ×	~5 ×	
MEGs expression level	0 ×	0 ×	0 ×	
Generation of UPD(14)pat	MR/PE	PE	TR/GC	
<Pregnancy and delivery>				
Polyhydramnios	13/13	1/1	9/9	23/23
Gestational age at Dx (w)	24 (19–30)	28	25.5 (14–27)	25 (14–30)
Amnioreduction	10/11	1/1	7/8	18/20
Amnioreduction (>30 w)	10/10	1/1	7/7	18/18
Placentomegaly[b]	7/10	1/1	6/6	14/17
Prenatal Dx of thoracic abnormality	2/11	0/1	6/8	8/20
Gestational age at Dx (w)	25 (22–28)	…	26 (23–33)	26 (22–33)
Prenatal Dx of abdominal abnormality	3/11	0/1	3/6	6/18
Gestational age at Dx (w)	25 (22–28)	…	26	26 (22–28)
Gestational age (w)	34 (24–37)	36	35.5 (28–38)	34.5 (24–38)
Premature delivery (<37 w)	11/13	1/1	5/9	17/23
Delivery (Caesarean:Vaginal)	12:1	0:1	3:6	15:8
Medically assisted reproduction	1/10	0/1	0/7	1/18
<Growth pattern>				
Prenatal growth failure[c]	0/13	0/1	0/9	0/23
Prenatal overgrowth[d]	8/13	0/1	5/9	13/23
Birth length (patient number)	12	1	8	21
SD score, median (range)	+0.2 (–1.2 ~ +3.0)	–0.1	+0.6 (–1.7 ~ +1.4)	+0.3 (–1.7 ~ +3.0)
Actual length (cm), median (range)	45.1 (30.6 ~ 51.0)	46.0	46.0 (36.0 ~ 50.0)	45.0 (30.6 ~ 51.0)
Birth weight (patient number)	13	1	9	23
SD score, median (range)	+2.4 (0.2 ~ +8.8)	+1.8	+2.2 (+0.1 ~ +3.2)	+2.2 (+0.1 +8.8)
Actual weight (cm), median (range)	2.79 (1.24 ~ 3.17)	2.94	2.56 (1.61 ~ 3.77)	2.79 (1.24 ~ 3.77)
Postnatal growth failure[e]	4/12	0/1	3/7	7/20
Postnatal overgrowth[f]	0/12	0/1	1/7	1/20
Present stature (patient number)	12	1	7	20
SD score, median (range)	–1.4 (–8.7 ~ 0.8)	1.1	–1.9 (–5.0 ~ +0.6)	–1.6 (–8.7 ~ +1.1)
Present weight (patient number)	12	1	7	20
SD score, median (range)	–1.2 (–6.0 ~ +1.4)	+1.2	–1.0 (–1.9 ~ +2.4)	–1.0 (–6.0 ~ +2.4)
<Craniofaciocervical features>				
Frontal bossing	11/12	0/1	6/9	17/22
Hairy forehead	10/12	1/1	7/9	18/22
Blepharophimosis	10/12	1/1	7/9	18/22
Small ears	6/12	0/1	2/8	8/21
Depressed nasal bridge	13/13	1/1	9/9	23/23
Anteverted nares	11/12	0/1	8/9	19/22
Full cheek	11/11	1/1	8/9	20/21
Protruding philtrum	13/13	1/1	9/9	23/23

Puckered lips	6/12	0/1	5/8	11/21
Micrognathia	12/12	1/1	7/8	20/21
Short webbed neck	13/13	1/1	8/8	22/22
<Thoracic abnormality>				
Small bell-shaped thorax in infancy[g]	13/13	1/1	9/9	23/23
Coat-hanger appearance in infancy[h]	13/13	1/1	9/9	23/23
Laryngomalacia	3/11	1/1	4/8	8/20
Tracheostomy	4/12	0/1	3/8	7/21
Mechanical ventilation	13/13	0/1	8/9	21/23
Duration of ventilation (m)[i]	1.2 (0.1–12)	…	1.2 (0.2–17)	1.2 (0.1–17)
<Abdominal wall defects>				
Omphalocele	5/13	0/1	2/9	7/23
Diastasis recti	8/13	1/1	7/9	16/23
<Developmental delay>				
Developmental delay	12/12	1/1	8/8	21/21
Developmental/intellectual quotient	58.5 (29–70)	46	44.5 (32–60)	55 (29–70)
Delayed head control (> 4 m)[j]	9/10	0/1	5/5	14/16
Age at head control (m)[k]	7 (3–36)	4	9 (6–14)	7 (3–36)
Delayed sitting without support (> 7 m)[j]	10/10	1/1	5/5	16/16
Age at sitting without support (m)[k]	12 (9–19)	10	13 (8–25)	12 (8–25)
Delayed walking without support (>14 m)[j]	10/10	1/1	6/6	17/17
Age at walking without support (m)[k]	25 (22–33)	23	27 (20–49)	25.5 (20–49)
<Other features>				
Feeding difficulty	12/12	1/1	7/8	20/21
Duration of tube feeding (m)[l]	6 (0.1–36)	0.6	39 (6−72)	6 (0.1–72)
Joint contractures	8/13	1/1	5/8	14/22
Constipation	6/12	1/1	5/7	12/20
Kyphoscoliosis	4/12	1/1	4/8	9/21
Coxa valga	5/12	0/1	1/8	6/21
Cardiac disease	4/13	0/1	1/8	5/22
Inguinal hernia	4/13	0/1	1/8	5/22
Seizure	1/12	0/1	0/8	1/21
Hepatoblastoma	1/13	0/1	2/9	3/23
<Mortality within the first five years>				
Alive:Deceased	11:2	1:0	6:3	18:5
<Parents>				
Paternal age at childbirth (y)	34.5 (26–38)	24	35 (26–47)	35 (24–47)
Maternal age at childbirth (y)	33 (26-42)	27	29 (25–43)	31 (25–43)
Advanced childbearing age (≥35 y)	6/13	0/1	2/9	8/23

MR: monosomy rescue; PE: postzygotic mitotic error; TR: trisomy rescue; and GC: gamete complementation.

[a–l] See the legends for Table 1, regarding their explanations.

Table S4. Clinical features in Kagami-Ogata syndrome (KOS) (UPD(14)pat and related conditions) and Beckwith-Wiedemann syndrome (BWS)

	KOS[a]	BWS[b]
**Clinical features identified in both KOS and BWS (at least one patient)**		
Polyhydramnios	97.0%	+
Placentomegaly	85.1%	+
Premature delivery (<37 w)	79.4%	+
Prenatal overgrowth (Macrosomia)	58.8%	+ (50.5%)
Postnatal overgrowth	6.6%	+
Abdominal wall defects	100.0%	+ (51.6%)
Omphalocele	32.3%	+
Diastasis recti	67.6%	+
Cardiac anomalies/cardiomyopathy	24.2%	+
Abdominal organomegaly	2.9%	+
Renal abnormalities	2.9%	+
Hepatoblastoma	8.8%	+ (12.5%)[c]
Developmental delay	100.0%	+[d]
**Clinical features observed only in KOS**		
Craniofaciocervical features		
Frontal bossing	75.7%	
Hairy forehead	69.6%	
Blepharophimosis	72.7%	
Small ears	37.5%	
Depressed nasal bridge	94.1%	
Anteverted nares	84.8%	
Full cheeks	93.1%	
Protruding philtrum	97.0%	
Puckered lips	53.1%	
Micrognathia	93.7%	
Short webbed neck	100.0%	
Thoracic abnormality		
Small bell-shaped thorax	100.0%	
Coat-hanger appearance	100.0%	
Laryngomalacia	41.3%	
Tracheostomy	34.4%	
Mechanical ventilation	94.1%	
Feeding difficulty	96.7%	
**Clinical features observed only in BWS**		
Craniofaciocervical features		
Anterior ear lobe crease and/or posterior helical pits		+ (48.0%)
Cleft palate		+
Macroglossia		+ (67.7%)
Advanced bone age		+
Neonatal hypoglycemia		+ (37.6%)
Nevus flammeus		+
Hemihyperplasia		+ (24.3%)
Cytomegaly of adrenal fetal cortex		+
Other embryonal tumors (Wilms tumor, neuroblastoma etc.)		+ (12.5%)[c]

BWS is caused by UPD(11)pat (mosaic) (16%), hypermethylation of the *H19* -DMR (9%), hypomethylation of the KvDMR1 (44%), loss-of-function mutations of *CDKN1C* (5%), and other unknown factors (25%).[1]

[a] Based on this study (see Supplementary Table S2 for details).

[b] Based on Weksberg *et al.*[2] and DeBaun *et al.*[3]

[c] The total frequency of hepatoblastoma, Wilms tumor, and neuroblastoma.

[d] Often manifested by patients with UPD(11)pat (mosaic).

References

1. Sasaki K, Soejima H, Higashimoto K *et al* : Japanese and North American/European patients with Beckwith-Wiedemann syndrome have different frequencies of some epigenetic and genetic alterations. *Eur J Hum Genet* 2007; **15:** 1205-1210.
2. Weksberg R, Shuman C, Beckwith JB: Beckwith-Wiedemann syndrome. *Eur J Hum Genet* 2010; **18:** 8-14.
3. DeBaun MR, Niemitz EL, McNeil DE, Brandenburg SA, Lee MP, Feinberg AP: Epigenetic alterations of H19 and LIT1 distinguish patients with Beckwith-Wiedemann syndrome with cancer and birth defects. *Am J Hum Genet* 2002; **70**: 604-611.

Table S5. Clinical manifestations in 17 non-Japanese patients reported in the literature

	UPD(14)pat	Microdeletions	
		Subtype 2	Subtype 4
	Pts 35–48 (n=14)	Pts 49–50 (n=2)	Pt 51 (n=1)
Age at the last examination or death (y)	0.5 (0.1–9.0)	5.2 (4.8-5.5)	2.3
Sex (male:female)	5:9	1:1	1:0
<Molecular findings>			
IG-DMR of maternal origin	Absent	Unmethylated	Unmethylated
MEG3-DMR of maternal origin	Absent	Deleted	Deleted
DLK1 expression level	2 ×	2 × (1 ×)	2 × (1 ×)
RTL1 expression level	~5 ×	~5 × (1 × or ~2.5 ×)	~2.5 × (~2.5 ×)
MEGs expression level	0 ×	0 × (1 × or 0 ×)	0 ×(0 ×)
<Pregnancy and delivery>			
Polyhydramnios	12/12	2/2	1/1
Gestational age at Dx (w)	28 (25–30)	Unknown	Unknown
Amnioreduction	3/4	Unknown	Unknown
Amnioreduction (>30 w)	2/3	Unknown	Unknown
Placentomegaly	1/1	Unknown	0/1
Prenatal Dx of thoracic abnormality	1/1	Unknown	Unknown
Gestational age at Dx (w)	23	Unknown	Unknown
Prenatal Dx of abdominal abnormality	1/1	Unknown	Unknown
Gestational age at Dx (w)	26	Unknown	Unknown
Gestational age (w)	33 (28–38)	35 (35)	32
Premature delivery (<37 w)	11/13	2/2	1/1
Delivery (Caesarean:Vaginal)	5:5	1:1	0:1
Medically assisted reproduction	N.D.	Unknown	Unknown
<Growth pattern>			
Prenatal growth failure	1/8	0/2	0/1
Prenatal overgrowth	0/8	0/2	0/1
Postnatal growth failure	1/2	Unknown	0/1
Postnatal overgrowth	0/2	Unknown	0/1
<Craniofaciocervical features>			
Frontal bossing	8/8	2/2	Unknown
Hairy forehead	6/8	Unknown	Unknown
Blepharophimosis	10/11	2/2	Unknown
Small ears	8/8	Unknown	Unknown
Depressed nasal bridge	10/10	2/2	Unknown
Anteverted nares	6/7	2/2	Unknown
Full cheeks	N.D.	2/2	Unknown
Protruding philtrum	11/11	2/2	Unknown
Puckered lips	2/3	Unknown	Unknown
Micrognathia	9/10	2/2	1/1
Short webbed neck	9/10	2/2	Unknown
<Thoracic abnormality>			
Small bell-shaped thorax in infancy	11/11	2/2	1/1
Coat-hanger appearance in infancy	10/10	2/2	1/1
Laryngomalacia	1/1	Unknown	Unknown
Tracheostomy	3/5	Unknown	+
Mechanical ventilation	10/11	1/2	1/1
Duration of ventilation (m)	0.8 (0–1.5)	0.5	3
<Abdominal wall defect>			
Omphalocele	1/11	0/2	Unknown
Diastasis recti	10/11	1/2	Unknown
<Developmental delay>			
Developmental delay	9/9	1/1	1/1
Developmental/intellectual quotient	Unknown	Unknown	Unknown

Delayed head control (> 4 m)	Unknown	Unknown	Unknown
Age at head control (m)	Unknown	Unknown	Unknown
Delayed sitting without support (> 7 m)	Unknown	Unknown	Unknown
Age at sitting without support (m)	Unknown	Unknown	Unknown
Delayed walking without support (>14 m)	Unknown	Unknown	+
Age at walking without support (m)	Unknown	Unknown	27
<Other features>			
Feeding difficulty	8/8	1/2	1/1
Duration of tube feeding (m)	Unknown	12	On going
Joint contractures	9/10	2/2	Unknown
Constipation	Unknown	Unknown	Unknown
Kyphoscoliosis	5/5	Unknown	Unknown
Laryngomalacia	1/1	Unknown	Unknown
Coxa valga	2/2	Unknown	Unknown
Cardiac disease	6/8	Unknown	Unknown
Inguinal hernia	2/3	1/2	Unknown
Seizure	2/3	Unknown	1/1
Hepatoblastoma	Unknown	Unknown	Unknown
<Mortality within the first five years>			
Alive:Deceased	4:8	2:0	1:0
<Parents>			
Paternal age at childbirth (y)	25 (15–34)	Unknown	Unknown
Maternal age at childbirth (y)	26 (13–37)	Unknown	Unknown
Advanced childbearing age (≥35 y)	1/10	…	…
References	1–14	15	15

The Irish Patient #32 is not included, because detailed clinical finding of Patient #32 are shown in Supplemental Table S2.

For molecular findings, see Supplementary Figure S1.

See also the legends for Table 1.

y: year; m: month; w: week; Dx: diagnosis; and N.D.: not determined.

See Supplementary Table S1 for references.

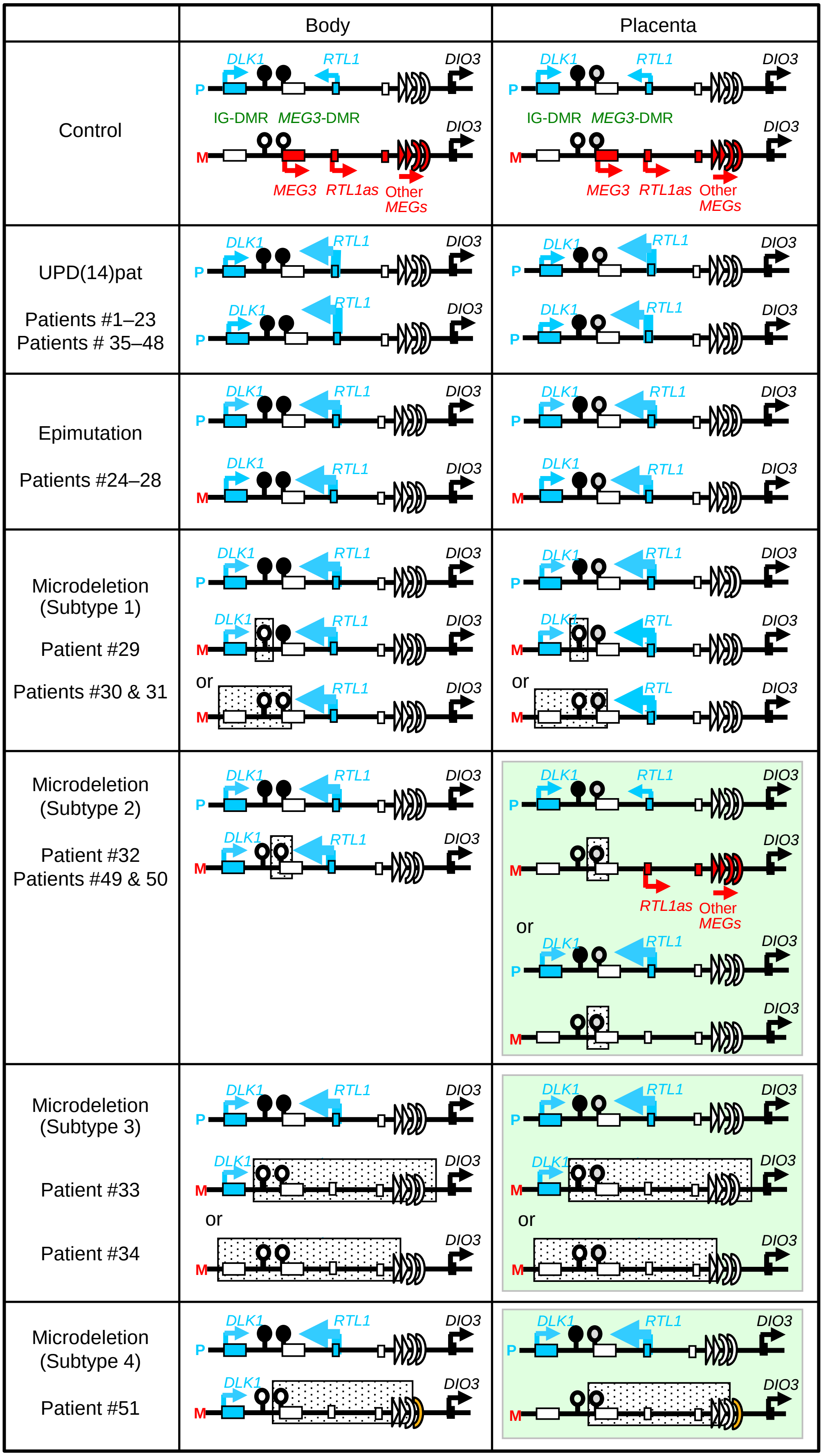
Body
Placenta
Control
DLK1
RTL1
DIO3
P
IG-DMR
MEG3-DMR
M
MEG3
RTL1as
Other MEGs
UPD(14)pat
Patients #1–23
Patients # 35–48
Epimutation
Patients #24–28
Microdeletion (Subtype 1)
Patient #29
Patients #30 & 31
or
Microdeletion (Subtype 2)
Patient #32
Patients #49 & 50
Microdeletion (Subtype 3)
Patient #33
Patient #34
Microdeletion (Subtype 4)
Patient #51

Supplementary Figure S1. Schematic representation of the observed and predicted methylation patterns of the DMRs and the expression patterns of the imprinted genes at human chromosome 14q32.2. Patients #1–34 are described in this report, whereas patients #35–51 are derived from the literature (see Supplementary Table S1). The placental data highlighted with light green are predicted findings, and the remaining data are observed findings. This figure has been constructed on the basis of our previous data.[1-3] P: paternally derived chromosome; and M: maternally derived chromosome. Filled and open circles represent hypermethylated and hypomethylated DMRs, respectively; since the *MEG3*-DMR is grossly hypomethylated and regarded as non-DMR in the placenta, it is painted in gray. Expressed *PEGs* (*DLK1* and *RTL1*) are shown in blue, expressed *MEGs* (*MEG3*, *RTL1as*, *MEG8*, *snoRNAs*, and *miRNAs*) in red, non-expressed genes in white, and a probably non-imprinted gene (*DIO3*) in black. Thick arrows for *RTL1* represent ~2.5 times increased *RTL1* expression that is ascribed to loss of functional microRNA-containing *RTL1as* as a repressor for *RTL1*. Loss or epimutation (hypermethylation) of the maternally derived *MEG3*-DMR results in maternal to paternal epigenotypic alteration in the body, whereas that of the maternally inherited IG-DMR leads to maternal to paternal epigenotypic alteration in the placenta as well as in the body because of epimutation (hypermethylation) of the *MEG3*-DMR (the IG-DMR behaves hierarchically as an upstream regulator for the methylation pattern of the *MEG3*-DMR in the body but not in the placenta). For microdeletions, the deleted regions are indicated by stippled rectangles. In patient #29, loss of the IG-DMR of maternal origin leads to methylation of the *MEG3*-DMR in the body, but not in the placenta. Since the microdeletion subtype 2 (patient #32, #49, and #50) removes the *MEG3*-DMR at the *MEG3* promoter region and three of the seven *MEG3* exons, *MEG3* expression is predicted to be absent. Other *MEGs* are predicted to be expressed from the maternally derived chromosome 14 containing the hypomethylated IG-DMR, while it may also be possible that all the *MEGs* are splice variants derived from a long transcript, so that loss of the *MEG3*-DMR affects expression of all the *MEGs*. Similarly, it also remains to be determined whether several *miRNAs* (shown in orange) in non-Japanese patient #51 (subtype 4) reported in the literature[4] (Supplementary Table S5) are transcribed from the maternally derived chromosome 14 containing the IG-DMR or remain silenced because of loss of the *MEG3*-DMR.

References

1. Kagami M, Sekita Y, Nishimura G *et al*: Deletions and epimutations affecting the human 14q32.2 imprinted region in individuals with paternal and maternal upd(14)-like phenotypes. *Nat Genet* 2008; **40:** 237-242.
2. Kagami M, Matsuoka K, Nagai T *et al*: Paternal uniparental disomy 14 and related disorders: placental gene expression analyses and histological examinations. *Epigenetics* 2012; **7:** 1142-1150.
3. Kagami M, O'Sullivan MJ, Green AJ *et al*: The IG-DMR and the MEG3-DMR at human chromosome 14q32.2: hierarchical interaction and distinct functional properties as imprinting control centers. *PLoS Genet* 2010; **6:** e1000992.
4. Beygo J, Elbracht M, de Groot K *et al*: Novel deletions affecting the MEG3-DMR provide further evidence for a hierarchical regulation of imprinting in 14q32. *Eur J Hum Genet* [Epub ahead of print].

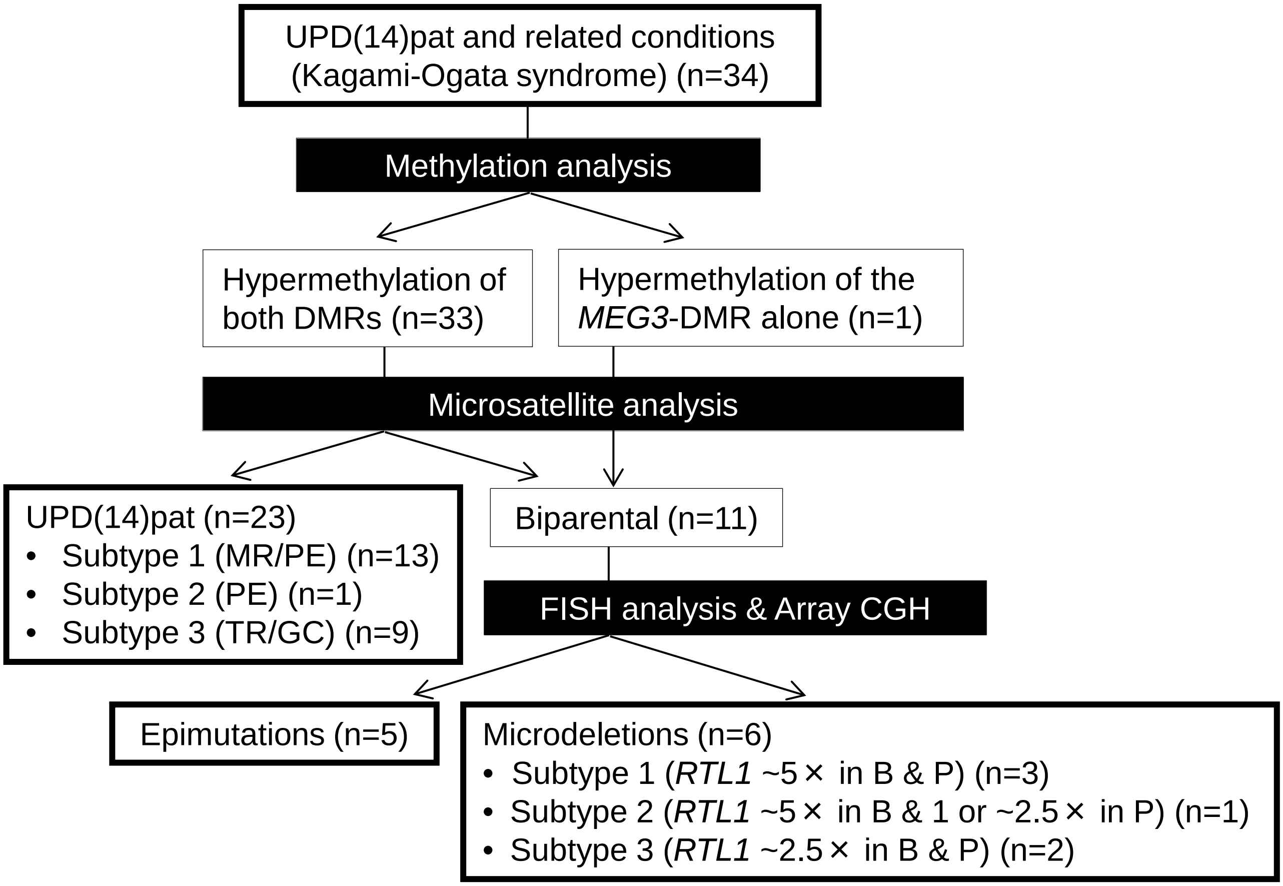

Supplementary Figure S2. Classification of 34 patients with UPD(14)pat and related conditions (Kagami-Ogata syndrome). MR: monosomy rescue; PE: post-fertilization mitotic error; TR: trisomy rescue; GC: gamete complementation; B: body; and P: placenta.

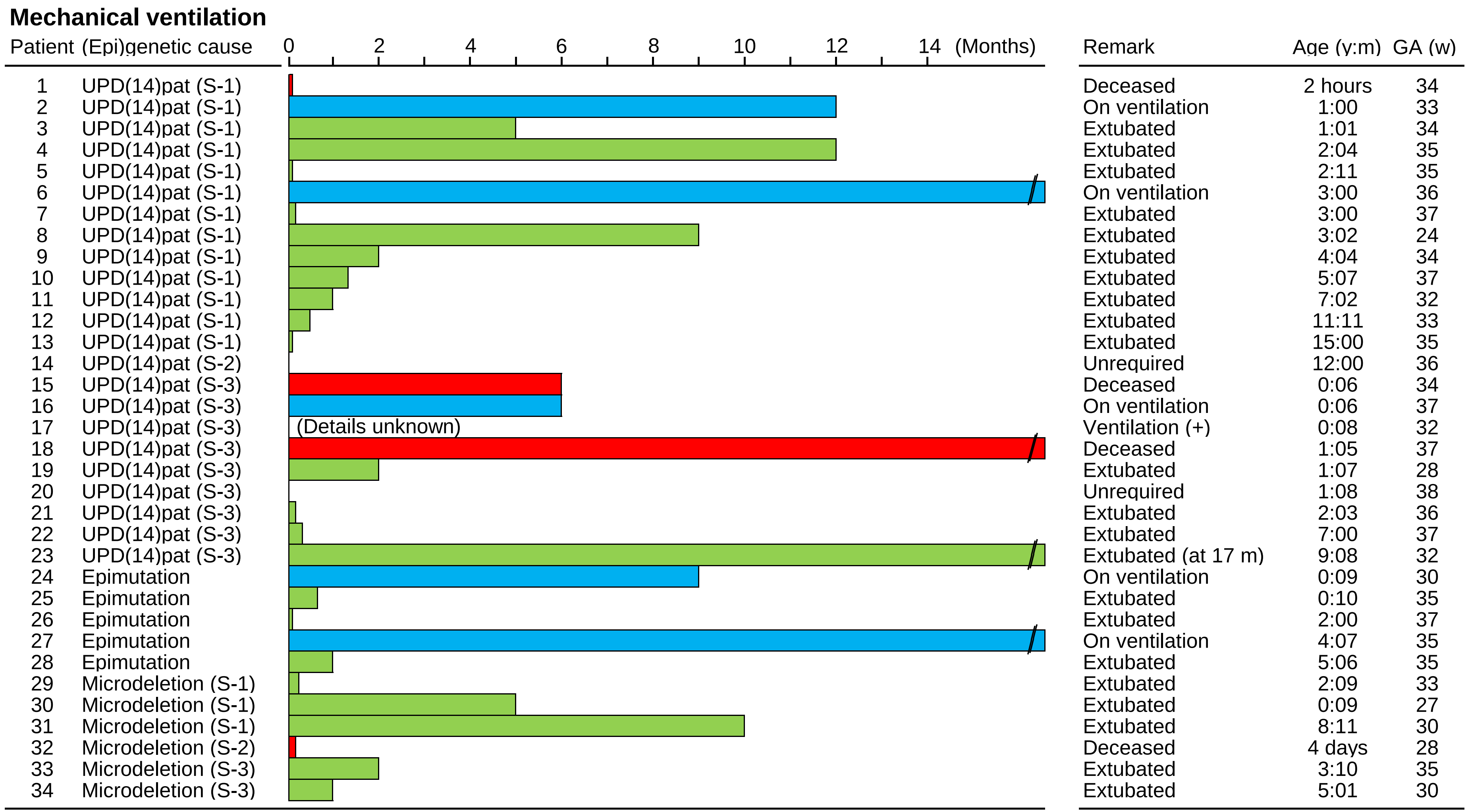

Supplementary Figure S3. The duration of mechanical ventilation. The green, blue, and red bars indicate the duration of ventilation in extubated, mechanically ventilated, and deceased patients, respectively. Age: age at the last examination or at death; and GA: gestational age.

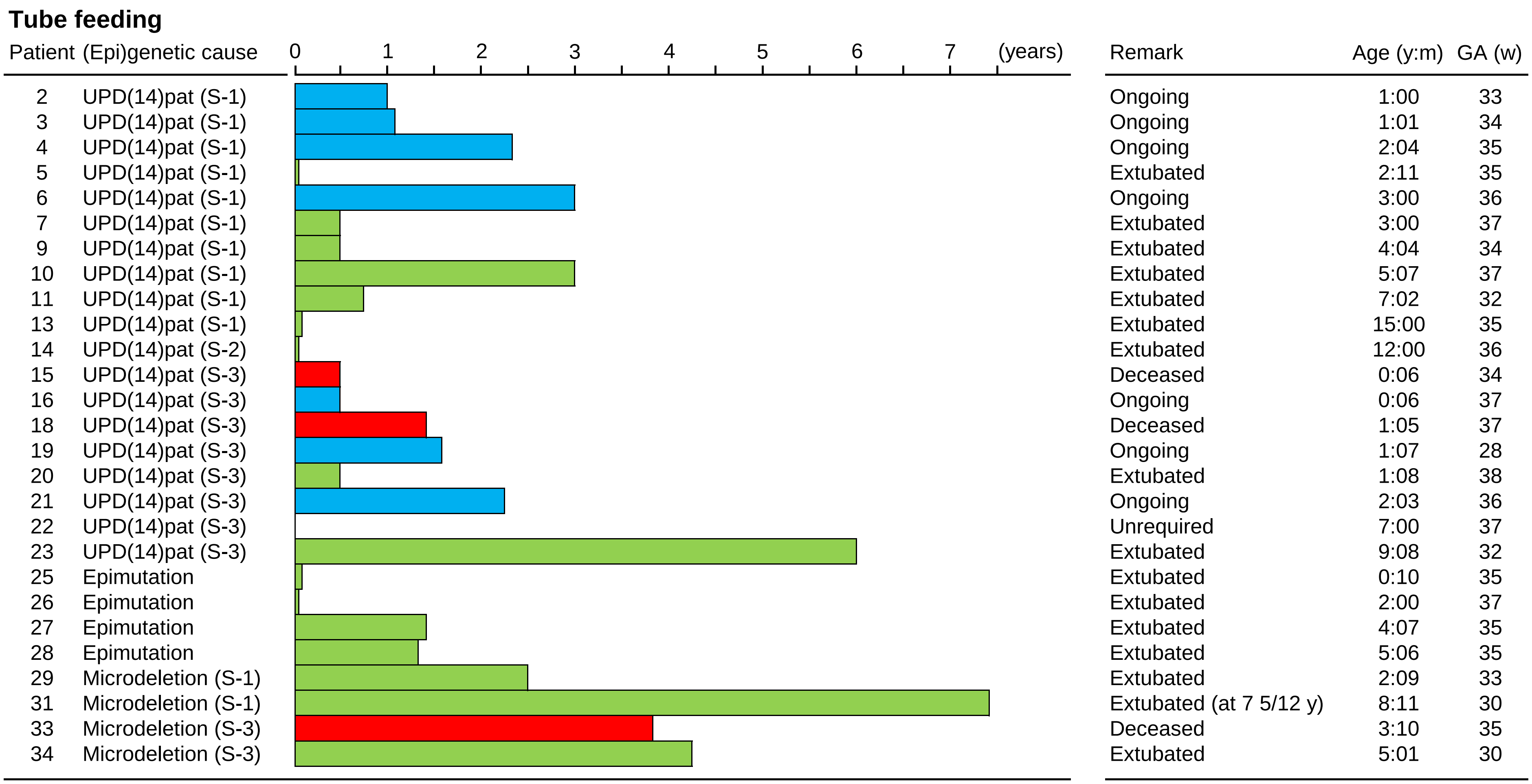

Supplementary Figure S4. The duration of tube feeding. The green, blue, and red bars indicate the duration of tube feeding in extubated patients, patients receiving tube-feeding, and deceased patients, respectively. Age: age at the last examination or at death; and GA: gestational age.

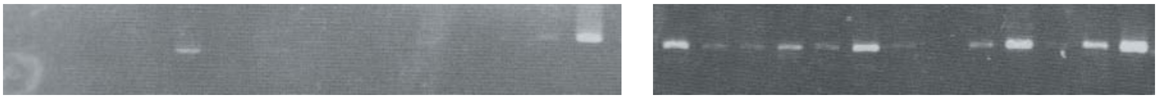

Supplementary Figure S5. *Rtl1* expression pattern in fetal mice at 18.5 days post coitus. To avoid concomitant amplification of *Rtl1as*, 3'-RACE was performed using 20 ng of mRNA extracted from each tissue, as reported previously.[1]

Reference

Sekita Y, Wagatsuma H, Irie M *et al*: Aberrant regulation of imprinted gene expression in Gtl2lacZ mice. *Cytogenet Genome Res* 2006; **113:** 223-229.
